# Association of sodium–glucose cotransporter-2 inhibitors with mortality across the spectrum of myocardial infarction: a systematic review and meta-analysis

**DOI:** 10.1186/s12933-025-02592-0

**Published:** 2025-01-22

**Authors:** Michele Maremmani, Ramin Ebrahimi, Marco Centola, Felice Achilli, Valentina Capone, Eduardo Bossone, Christian Templin, Davide Di Vece

**Affiliations:** 1 Cardiovascular and Thoracic Department, Pio XI Hospital, Desio, Italy; 2 Department of Internal Medicine B, University of Medicine Greifswald, Ferdinand-Sauerbruch-Straße, 17475 Greifswald, Germany; 3 First Clinic of Internal Medicine, Department of Internal Medicine, University of Genoa, Genoa, Italy; 4 Cardiology Division, Antonio Cardarelli Hospital, Naples, Italy; 5 Department of Advanced Biomedical Sciences, University of Naples Federico II, Naples, Italy; 6 Department of Public Health, University of Naples Federico II, Naples, Italy

**Keywords:** Sodium–glucose cotransporter-2 inhibitors, Myocardial infarction, Mortality, Diabetes, Meta-analysis

## Abstract

**Background:**

The impact of sodium–glucose cotransporter-2 (SGLT2) inhibitors on mortality following myocardial infarction (MI) remains uncertain. Additionally, the role of type 2 diabetes mellitus (T2DM) and heart failure (HF) in modulating the effectiveness of these drugs post-MI are not fully understood. This meta-analysis aimed to assess the association of SGLT2 inhibitors with all-cause mortality in post-MI patients and to explore key moderators influencing this benefit.

**Methods:**

PubMed, Embase, and Scopus were searched for randomized controlled trials (RTCs) and propensity score-matched (PSM) observational studies assessing SGLT2 inhibitors' impact on post-MI mortality. The primary outcome was all-cause mortality. We pooled hazard ratios (HRs) to estimate the intervention's effect on the overall population and stratified studies into early (SGLT2 inhibitors administered within eight weeks post-MI) and delayed treatment trials. Meta-regression assessed the moderating effects of T2DM and HF.

**Results:**

A total of five RCTs and four PSM observational studies involving 26,753 patients (mean [SD] age, 62.9 [10.5] years; 6,406 female [24.0%]; 16,369 T2DM [61.2%]; 13,933 HF [52.1%]) were included. Early and delayed treatment trials comprised 16,165 (60.4%) and 10,588 (39.6%) patients, respectively. SGLT2 inhibitors reduced all-cause mortality following MI (HR 0.79, 95% CI [0.68, 0.91]; p = 0.001; I^2^ = 59%). Stratified analysis demonstrated consistent effects in both early (HR 0.76, 95% CI [0.59, 0.98]; p = 0.03; I^2^ = 65%) and delayed (HR 0.81, 95% CI [0.67, 0.98]; p = 0.03; I^2^ = 60%) treatment. Meta-regression identified T2DM as a significant moderator of the mortality benefit (β = − 0.0049; p = 0.0006).

**Conclusion:**

In this meta-analysis, early and delayed treatment with SGLT2 inhibitors following MI was associated with a significant reduction in all-cause mortality*.* Furthermore, the presence of T2DM was associated with a greater mortality reduction, while HF was not significantly associated with the outcome.

**Graphical Abstract:**

Association of SGLT2 Inhibitors with Mortality Across the Spectrum of Myocardial Infarction. Data from 26,753 post-MI patients are summarized, including baseline characteristics. The plots represent the pooled hazard ratios (HRs) with 95% confidence intervals (CIs), comparing SGLT2 inhibitors to control (placebo/no treatment), with HRs below 1 favoring SGLT2 inhibitors. The diagram shows early and delayed treatment trial subgroups, presenting the number of participants, the percentage receiving SGLT2 inhibitors, and the respective HRs for mortality. The meta-regression panel highlights T2DM and HF as moderators, reporting β-coefficients (β), p-values, and residual heterogeneity (I^2^). Negative β (−) indicates that as the percentage of the moderator increases, the HR for mortality decreases. Abbreviations: HF, heart failure; MI, myocardial infarction; SGLT2i, sodium–glucose cotransporter-2 inhibitors; T2DM, type 2 diabetes mellitus.
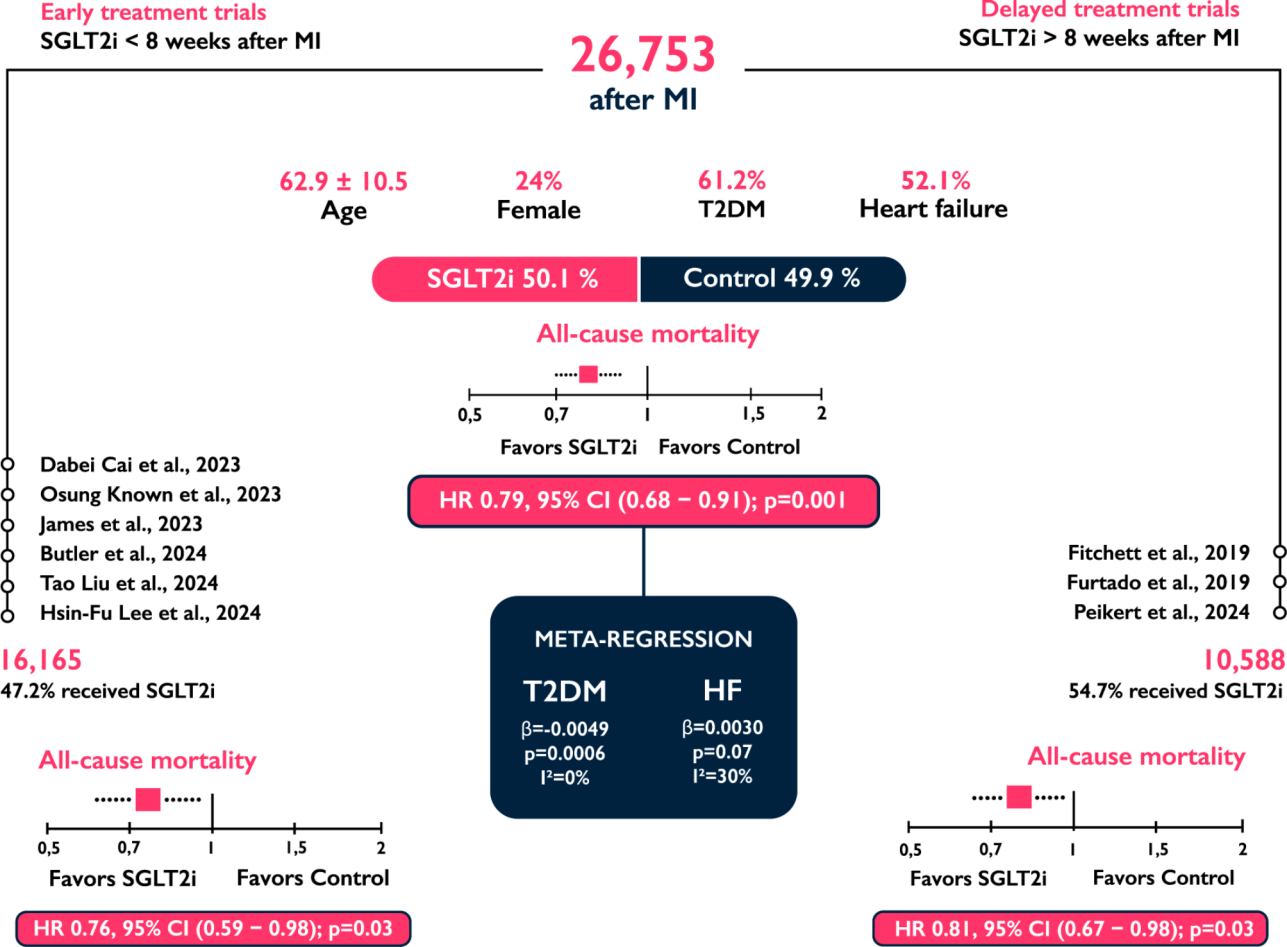

**Supplementary Information:**

The online version contains supplementary material available at 10.1186/s12933-025-02592-0.

## Introduction

Gliflozins, known as Sodium–glucose cotransporter-2 (SGLT2) inhibitors, have shown significant cardiovascular (CV) benefit in high-risk patients, particularly those with type 2 diabetes mellitus (T2DM) [1, 2], chronic kidney disease (CKD) [3–5], and heart failure (HF) across a wide range of left ventricular ejection fractions [6–10].

In patients with a history of myocardial infarction (MI), SGLT2 inhibitors have been shown to reduce major adverse cardiovascular events (MACEs) and hospitalization for HF (HHF) among those with T2DM [11, 12]. However, the evidence regarding their effectiveness in reducing mortality remains inconsistent, particularly for patients without T2DM [13]. This highlights the need for a comprehensive meta-analysis to provide clearer insights into this outcome.

Recent studies have also suggested pleiotropic effects of SGLT2 inhibitors in patients with acute MI(AMI) [14–17], leading to randomized controlled trials (RCTs), such as DAPA-MI [18] and EMPACT-MI [19], which investigated their safety and efficacy in this context [18, 19]. Early treatment with SGLT2 inhibitors following an AMI has been shown to be safe, to improve cardiometabolic health [18], as well as to reduce HHF rates in patients with risk factors such as T2DM, HF, and CKD [19]. Despite these benefits, both DAPA-MI [18] and EMPACT-MI [19] did not demonstrate a significant reduction in mortality with SGLT2 inhibitors. Notably, the DAPA-MI [18] excluded patients with T2DM and HF, while the EMPACT-MI [19] included a small proportion of patients with T2DM (approximately 30%) and a subset with signs or symptoms of HF. This heterogeneity may have contributed to the inconsistent impact on mortality across these trials. Conversely, several observational studies primarily involving patients with T2DM, have reported a significant reduction in mortality following AMI among those treated with SGLT2 inhibitors [20–26]. These findings suggest that the presence of T2DM is a critical factor in achieving mortality benefit for AMI patients receiving SGLT2 inhibitors. Therefore, the overall impact of SGLT2 inhibitors on mortality after MI, along with the role of key moderators, remains incompletely understood.

Given these complexities, the present meta-analysis aimed to assess the overall association of SGLT2 inhibitors with mortality in post-MI patients. To add granularity regarding the timing of SGLT2 inhibitor administration, we conducted a subgroup analysis, categorizing studies into early (administration within eight weeks after MI) and delayed treatment trials.

Additionally, meta-regression was employed to explore key moderators, such as the presence of T2DM and HF, to determine which subgroups derive the greatest mortality benefit. These analyses were designed to provide critical insights for optimizing the use of SGLT2 inhibitors in this population, addressing limitations in individual trials.

## Methods

### Study design

This systematic review and meta-analysis was conducted following the Preferred Reporting Items for Systematic Reviews and Meta-analysis (PRISMA) statement [27] and the Cochrane Collaboration guideline [28]. The prospective meta-analysis protocol was registered with the International Prospective Register of Systematic Reviews (PROSPERO, CRD42024546540). PRISMA checklists are presented in Tables S1 and S2 (Supplemental material online).

### Data source and search strategy

We systematically searched PubMed, Embase, and Scopus databases from inception through the final search date of May 15, 2024. We also used backward snowballing (ie, review of references and related articles sections) to identify relevant texts from articles identified in the original search. Two authors (M.M., and D.DV.) performed the systematic review independently, and disagreements were resolved by consensus. Study selection involved screening titles and abstracts followed by a full-text evaluation of potentially eligible studies. The complete search strategy for each database is presented in Table S3 (Supplemental material online).

### Eligibility criteria

For inclusion, no restrictions were determined concerning the publication date, status, or language. Eligible studies included RCTs and PSM observational studies, comparing SGLT2 inhibitors with control (placebo/no treatment) on mortality following MI with ≥ 12 months follow-up. Studies were excluded if they reported only surrogate endpoints, lacked propensity score-matching, or had follow-up < 12 months. Further details on trial designs are available in Table S4 (Supplemental material online).

### End points

Our pre-specified primary endpoint was all-cause mortality. A secondary post-hoc analysis was conducted to evaluate hospitalization for heart failure (HHF).

### Data extraction and quality assessment

Trial eligibility and data extraction were performed by two independent reviewers (M.M., D.DV.), and any disagreement was resolved by consensus. Study quality (performed by M.M., D.DV.) was evaluated using the Cochrane Risk of Bias Tool for RCTs [29, 30] and ROBINS-I tool [31] for observational studies.

### Subgroup stratification

Clinical trials of SGLT2 inhibitors and CV outcomes have randomized participants to treatment or placebo no earlier than eight weeks after MI [1, 2]. As a result, in clinical practice, SGLT2 inhibitors are usually initiated after eight weeks post-MI. For the purpose of this analysis, studies were classified as early treatment trials if SGTL2 inhibitors were administered within eight weeks following MI. Otherwise, they were categorized as delayed treatment trials.

### Statistical analysis

The primary analysis was conducted on the total population from the nine identified trials, with subgroup analyses stratified by early and delayed treatment trials and study design (RCTs and PSM studies). The primary outcome was all-cause mortality, with hazard ratios (HRs) and 95% confidence intervals (CIs) pooled using an inverse variance weighted random-effects model as implemented in the meta and metafor packages in R (Version 4.1.1). The secondary outcome was hospitalization for HF. Publication bias was assessed by visual inspection of the funnel plot; Egger's test was not feasible due to the small number of studies. Heterogeneity was evaluated using the Cochrane Q test and I^2^ statistic [32], with I^2^ thresholds of < 25%, 25–75%, and > 75% indicating low, moderate, and high heterogeneity, respectively. Substantial heterogeneity (I^2^ > 50%) prompted a meta-regression using a mixed-effects model to explore potential moderators, reporting τ^2^, I^2^, R^2^, β-coefficients, and p-values. A leave-one-out sensitivity analysis was conducted to test the robustness of results, using the metainf function in R. Descriptive statistics were reported as mean ± SD or median, with comparisons between groups made using t-tests for continuous variables and chi-squared tests for proportions. A two-sided p-value < 0.05 was considered statistically significant.

### Data sharing statement

All the data presented in this manuscript are derived from the analysis of previously published data, available in the original manuscripts and their respective supplemental appendices. All patients provided written informed consent before participation in individual RCTs, and the institutional review board approved the protocols at each site.

## Results

### Study selection

**Fig. 1:**
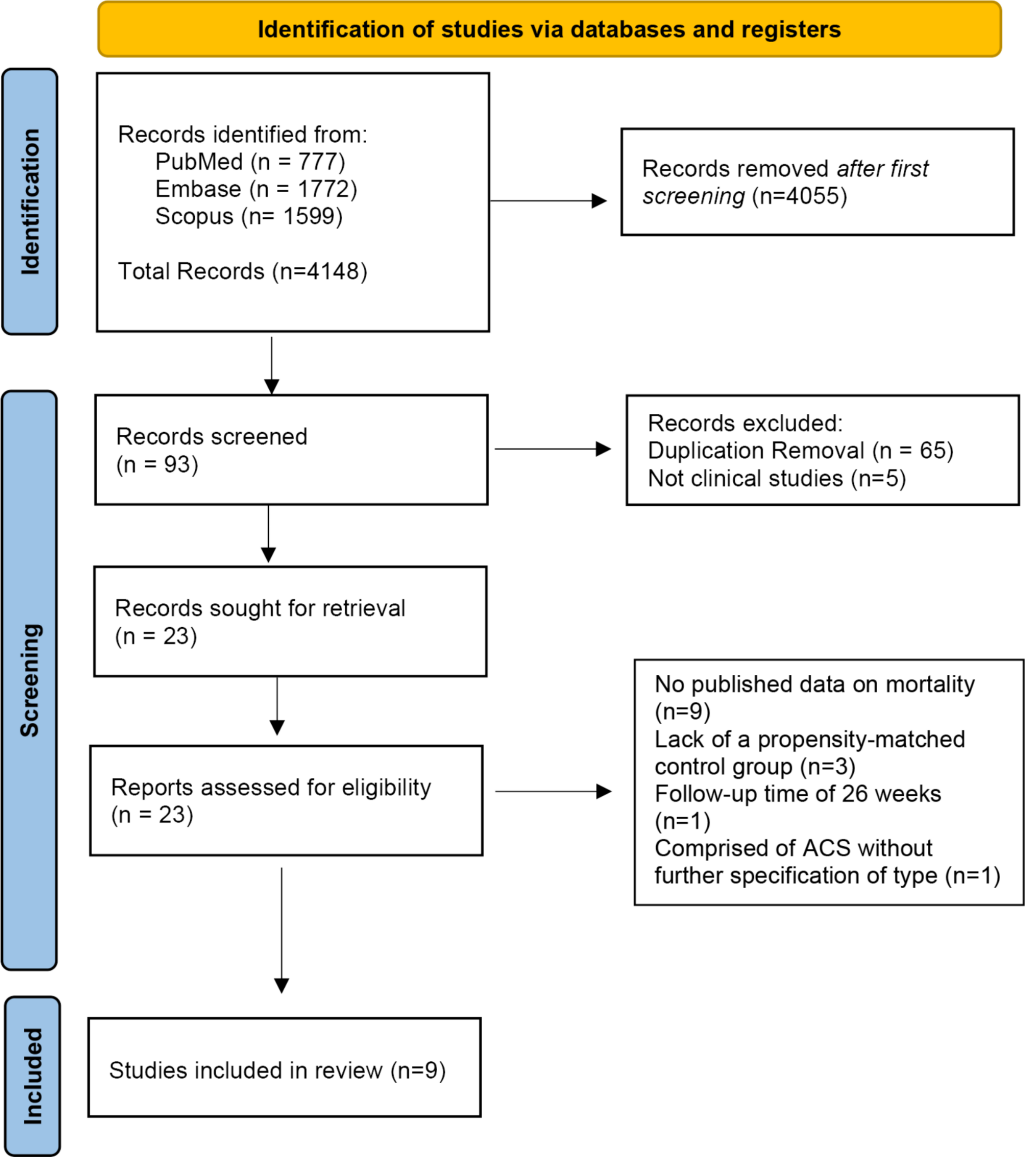
Search and study selection. The Preferred Reporting Items for Systematic Review and Meta-analysis flow-chart of studies included in the meta-analysis

The search strategy, conducted from inception until May 15th, 2024, yielded 4148 articles. Following the initial screening, 4055 articles were removed. Subsequently, 93 records were reviewed in full text. Ultimately, nine studies met the pre-specified inclusion criteria and were included in the analysis. The search strategy and PRISMA flowchart are summarized in Fig. 1.

This meta-analysis comprised two RCTs [18, 19], a pre-specified subgroup analysis of three clinical outcome RTCs [11–13], and four PSM observational studies [20–23], totaling 26,753 subjects.

The main characteristics of the studies, including study type, timing of SGLT2 inhibitors initiation post-MI, percentages of patients with HF and T2DM, and sample sizes for intervention and control groups are presented in Table 1*.* The studies are stratified by early and delayed treatment trials, as previously reported.

**Table 1:** Summary of main characteristics of studies included in the present meta-analysis

Included studies	Population	Study Type	SGLT2i timing Post-MI	Intervention	Heart failure (%)	Type 2 diabetes mellitus (%)	Intervention/Control (n)	Median FU (Months)	SGLT2i efficacy (95% CIs) for all-cause mortality
Early treatment trials									
Butler et al. [19] EMPACT-MI	AMI patients at risk for HF	Randomized controlled trial‡	Within 14 days following AMI	Empagliflozin 10 mg	56.9	31.9	3260/3262	17.9	HR 0.96 (0.78–1.19)
James et al. [18] DAPA-MI	AMI patients without T2DM or HF	Randomized controlled trial‡	Within 7–10 days following AMI	Dapagliflozin 10 mg	0	0	2019/1998	11.6	HR 1.22 (0.77–1.92)
Osung Known et al. [23]	AMI patients with T2DM treated with PCI	Observational with 1:2 propensity score matching†	Within 14 days following PCI for AMI	Dapagliflozin 10 mg (64.5%) Empagliflozin 10 mg (32.2%) Ipragliflozin 10 mg (3.3%)	2.8	100	938/1876	25.2	HR 0.55 (0.37–0.80)
Dabei Cai et al. [22]	AMI patients treated with PCI with T2DM or HF	Observational with 1:1 propensity score matching†	During hospitalization for AMI	Dapagliflozin 10 mg	15.9	93.6	236/236	23.8	HR 0.33 (0.13–0.82)
Hsin-Fu Lee et al. [20]	AMI patients with T2DM	Observational with 1:1 propensity score matching†	Within 12 weeks following discharge for AMI	Empagliflozin 10 mg (57%) Dapagliflozin 10 mg (38%) Canagliflozin 100 mg (5%)	4.5	100	944/944	23.4	HR 0.69 (0.50–0.95)
Tao Liu et al. [21]	T2DM patients hospitalized for ACS	Observational with 1:1 propensity score matching†	During hospitalization for ACS	SGLT2i (not specified)	40.5	100	226/226	12	HR 0.763 (0.561–1.040)
Delayed treatment trials									
Furtado et al. [12] DECLARE-TIMI 58 Sub-analysis	Prespecified sub-group analysis of patients with previous MI and T2DM	Prespecified sub-group analysis of randomized controlled trial‡	After 8 weeks from AMI	Dapagliflozin 10 mg	21.5	100	1777/1807	50.4	HR 0.83 (0.67–1.03)
Peikert et al. [13] DAPA-HF and DELIVER Sub-analysis	Planned participant-level pooled analysis of patients with previous MI and HF	Prespecified sub-group analysis of randomized controlled trial‡	After 12 weeks from AMI	Dapagliflozin 10 mg	100	49.2	1830/1901	22.9	HR 0.92 (0.78–1.07)
Fitchett et al. [11] EMPA-REG Sub-analysis	Prespecified sub-group analysis of patients with previous MI and T2DM	Prespecified sub-group analysis of randomized controlled trial‡	After 8 weeks from AMI	Empagliflozin 10 or 25 mg	12.1	100	3273/2190	37.2	HR 0.65 (0.50–0.84)

n = number of participants

ACS, acute coronary syndrome; AMI, acute myocardial infarction; HF, heart failure; HR, hazard ratio; MI, myocardial infarction; PCI, percutaneous coronary intervention; SGLT2i, sodium–glucose cotransporter 2 inhibitors; T2DM, type 2 diabetes mellitus

^†^Control group was defined as non-users of SGLT2 inhibitors

^‡^ Control group received placebo as part of randomized placebo-controlled trials

### Patient population in the pooled cohort

The pooled cohort consisted of 26,753 patients, with 13,420 in the SGLT2 inhibitor arm and 13,333 in the control arm, which received either placebo or no treatment with SGLT2 inhibitors. The mean age was similar between the intervention and control arms (62.9 ± 10.5 vs. 63.0 ± 10.6 years, p = 0.25), with the majority of patients being male (76.1% vs. 76.0%, p = 0.73). The prevalence of T2DM (61.3% vs. 61.0%, p = 0.64) and HF (52.2% vs. 52.0%, p = 0.76) was also comparable between the two groups.

Among patients in the SGLT2 inhibitor arm, 7,623 (56.8%) received early treatment (within eight weeks after MI), while 5,797 (43.2%) had a history of MI and received delayed treatment (at least eight weeks following MI). Of those treated, 6,826 (50.9%) received dapagliflozin, and 6,290 (46.9%) received empagliflozin.

Baseline characteristics of the pooled population are presented in Table 2*.*

**Table 2:** Baseline demographics in pooled population

	SGLT2i arm (n = 13,420)	Control arm (n = 13,333)	p value
Age (years), mean (SD)	62.9 (10.5)	63.0 (10.6)	p = 0.25
Male gender, n (%)	10,219 (76.1)	10,128 (76.0)	p = 0.73
T2DM, n (%)	8230 (61.3)	8139 (61.0)	p = 0.64
Heart failure, n (%)	7002 (52.2)	6931 (52.0)	p = 0.76
Early treatment after MI, n (%)	7623 (56.8)	8542 (64.2)	p < 0.05
STEMI	4711 (35.1)	5221 (39.2)	
NSTEMI	1904 (14.2)	2322 (17.4)	
Non-specified ST-type MI	944 (7.0)	944 (7.1)	
Delayed treatment after MI, n (%)	5797 (43.2)	4791 (36.0)	p < 0.05
SGLT2i type, n (%)			
Dapagliflozin	6826 (50.9)		
Empagliflozin	6290 (46.9)		
Non-specified SGLT2i	257 (1.9)		
Canagliflozin	47 (0.35)		

Values are Mean ± SD, n = numbers,

% = proportion relative to the total number of patients within the corresponding arm

MI, myocardial infarction; SGLT2i, sodium–glucose cotransporter-2 inhibitors; T2DM, type 2 diabetes mellitus

### Patient population stratified by early and delayed treatment trials

Six studies, classified as early treatment trials, included 16,165 patients (60.4%), of whom 7,623 (47.2%) received early SGLT2 inhibitors following MI. In three studies [18, 19, 23], SGLT2 inhibitors were administered within 14 days following AMI or PCI for AMI; in two studies [21, 22], during hospitalization for AMI or ACS; in one study [20], within 12 weeks following discharge for AMI.

Three studies, classified as delayed treatment trials pooled 10,588 patients (39.6%), of whom 5,797 (54.7%) received SGLT2 inhibitor treatment no earlier than eight weeks after MI in two studies [11, 12] or 12 weeks in one study [13]. Baseline demographics for the early and delayed treatment trials sub-groups, categorized by intervention and control arms, are detailed in Table 3*.*

**Table 3:** Baseline demographics stratified by early and delayed treatment trial

Arm	Early treatment post-MI (n = 16,165)		p value	Delayed treatment post-MI (n = 10,588)		p value
	Intervention	Control		Intervention	Control	
Sample size (n)	7623	8542		5797	4791	
Age (years), mean (SD)	62.03 (11.38)	62.17 (11.46)	p = 0.4	64.60 (8.77)	62.63 (8.83)	p < 0.05
Male gender, n (%)	5918 (77.6)	6566 (76.8)	p = 0.2	4301 (74.2)	3572 (74.6)	p = 0.6
T2DM, n (%)	3362 (44.1)	4314 (50.5)	p < 0.05	4867 (84.0)	3825 (79.8)	p < 0.05
Heart failure, n (%)	4525 (59.4)	4510 (52.8)	p < 0.05	2477 (42.7)	2421 (50.5)	p < 0.05
Type of SGLT2i, n (%)	Dapagliflozin, 3219 (42.2) Empagliflozin, 4100 (53.8) Non-specified SGLT2i 257 (1.6) Canagliflozin, 47 (0.6)	None		Dapagliflozin, 3607 (62.2) Empagliflozin, 2190 (37.8) Non-specified SGLT2i, 0 (0) Canagliflozin, 0 (0)	None	

Values are Mean ± SD, n = numbers, % = proportion relative to the total number of patients within the corresponding arm

MI, myocardial infarction; SGLT2i, sodium–glucose cotransporter 2 inhibitor; T2DM, type 2 diabetes mellitus

The differences in T2DM proportions between the intervention and control arms can be attributed to individual study designs, such as the 1:2 propensity score-matching in Know et al. [23], and the 1:1:1 randomization in Fitchett et al. [11], both of which influenced the distribution of T2DM patients across the groups. These imbalances in T2DM and HF proportions were accounted for through meta-regression, providing a more accurate assessment of the true association of SGLT2 inhibitors with mortality.

### Primary endpoint

The primary outcome of interest was all-cause mortality. A total of 1,963 events of all-cause death occurred: 900 in the SGLT2 inhibitors group and 1,063 in the control group.

**Fig. 2:**
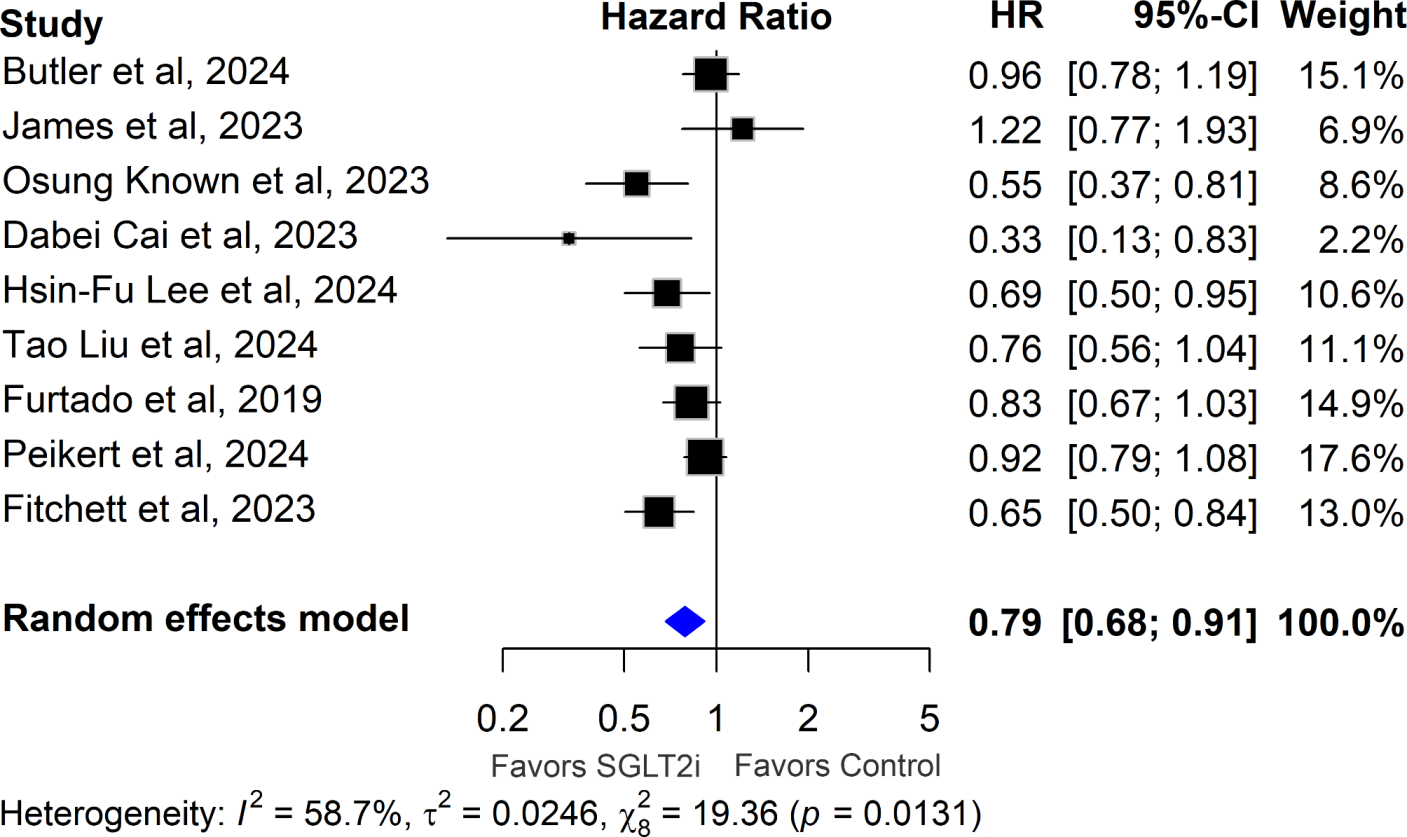
Association of SGLT2 Inhibitors with All-Cause Mortality after Myocardial Infarction. Hazard ratios (HRs) with 95% confidence intervals (CIs) are plotted, comparing SGLT2 inhibitors to control (placebo/no treatment) in post-MI patients. HRs below 1 indicate a reduction in all-cause mortality favoring SGLT2 inhibitors

Across the nine trials, median follow-up ranged from 11.6 months to 4.2 years. Overall, SGLT2 inhibitors significantly reduced the hazard of all-cause mortality (HR 0.79, 95% CI [0.68, 0.91]; p = 0.001; I^2^ = 59%), as demonstrated in Fig. 2.

**Fig. 3:**
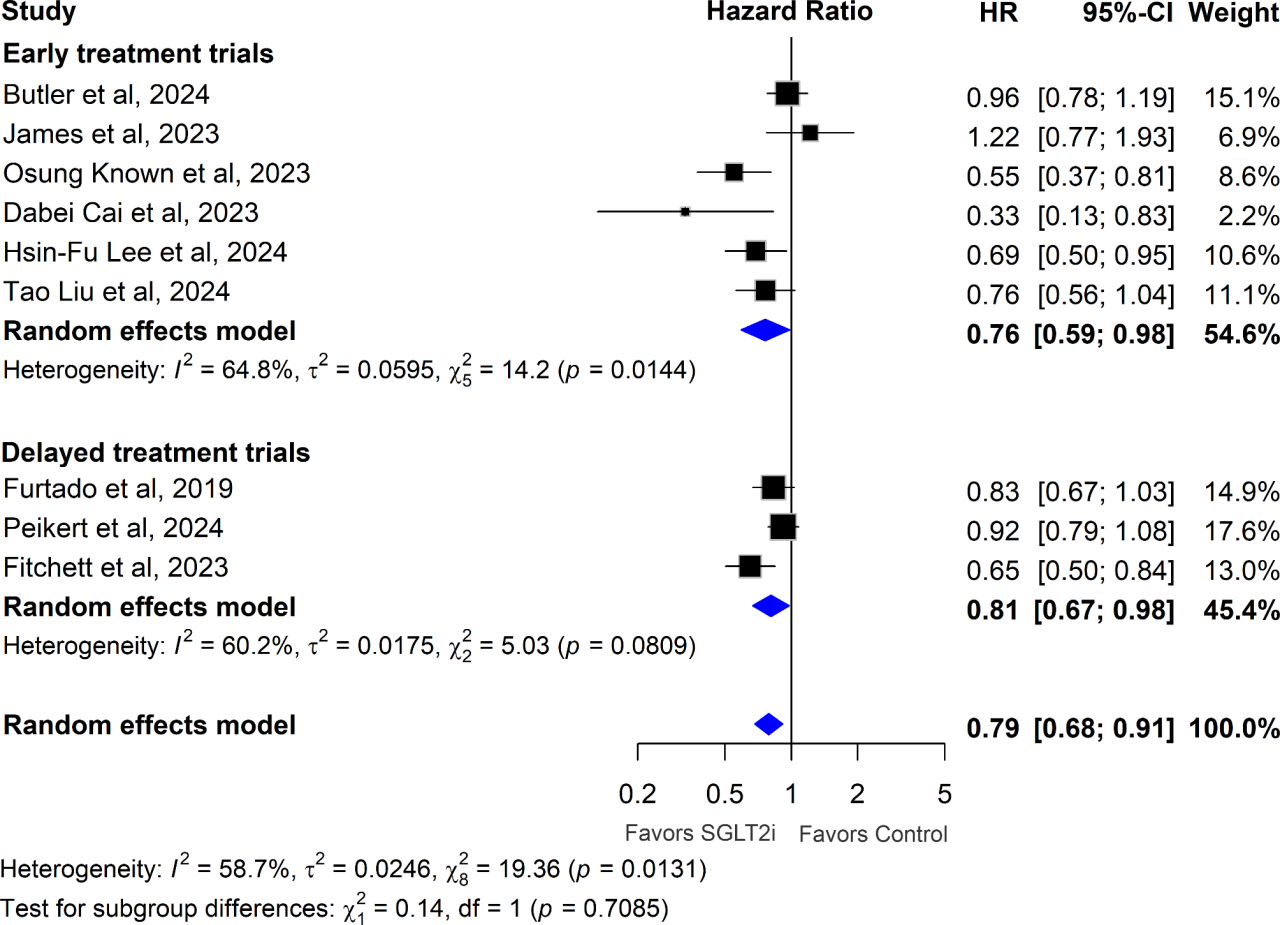
Association of SGLT2 Inhibitors with All-Cause Mortality: Analysis Stratified by Early and Delayed Treatment Trials. HRs with 95% CIs are plotted, comparing SGLT2 inhibitors to control in post-MI patients, stratified by early and delayed treatment trials. HRs below 1 indicate a reduction in all-cause mortality favoring SGLT2 inhibitors

Subgroup analysis indicated that early treatment with SGLT2 inhibitors following AMI significantly reduced the hazard of all-cause mortality (HR 0.76, 95% CI [0.59, 0.98]; p = 0.03; I^2^ = 65%), as demonstrated in Fig. 3. Similarly, delayed treatment with SGLT2 inhibitors in patients with a history of MI also resulted in a reduction in the hazard of all-cause mortality (HR 0.81, 95% CI [0.67, 0.98]; p = 0.03; I^2^ = 60%), as demonstrated in Fig. 3.

### Secondary endpoint

The secondary outcome of interest was HHF. Overall, SGLT2 inhibitors significantly reduced the hazard of HHF (HR 0.75, 95% CI [0.67, 0.83]; p = 0.001; I^2^ = 0%), as demonstrated in Fig. S1 (Supplemental Material Online).

### Sensitivity analysis

A leave-one-out sensitivity analysis was conducted to assess the robustness of the meta-analysis results for the primary endpoint in both the overall and subgroup analyses.

In the overall analysis, the results remained consistent when individual studies were excluded (Fig. S2, Supplemental material online). The HR ranged from 0.76 to 0.82, with p-values consistently below 0.01. Heterogeneity (I^2^) fluctuated between 53.0% and 63.8%, indicating that no single study significantly influenced the overall pooled results.

In the early treatment trials subgroup, the exclusion of certain studies, such as Tao Liu et al. [21], resulted in a non-significant HR (HR 0.74, 95% CI: [0.53; 1.05]). Despite this, the overall pooled effect was consistent across most analyses, demonstrating the robustness of the findings despite minor variations (Fig. S3, Supplemental material online).

In the delayed treatment trials subgroup, the exclusion of Fitchett et al. [11] led to a borderline non-significant HR (HR 0.88, 95% CI: [0.78; 1.00]), as shown in Fig. S4 (Supplemental material online). Despite this, the overall findings remained stable, with a trend toward reduced mortality across both subgroups. Although the exclusion of individual studies introduced minor variations in the pooled estimates, the overall findings consistently demonstrated a trend toward reduced mortality, reinforcing the robustness of the results across both subgroups.

For the secondary endpoint, leave-one-out sensitivity analysis showed consistent results across trials, with p-values remaining below 0.01 regardless of the study excluded (Fig. S5, Supplemental material online).

### Meta regression analysis

In the overall population, the presence of T2DM was significantly associated with a reduction in HR for mortality (β = − 0.0049, p = 0.0006), with each 1% increase in T2DM patients resulting in a 0.49% decrease in HR. Importantly, the model accounted for all heterogeneity between studies (R^2^ = 100%), confirming that T2DM is a key moderator of the mortality benefit observed with SGLT2 inhibitors. In contrast, HF was not a significant moderator of the effect of SGLT2 inhibitors on mortality (β = 0.0030, p = 0.0705), explaining a smaller portion of the between-study variability (R^2^ = 60%).

The subgroup meta-regression analysis of early treatment trials confirmed T2DM as a significant moderator in this population (β = − 0.0057, p = 0.0016, R^2^ = 99.9%), highlighting its pivotal role when SGLT2 inhibitors are administered early following an AMI. Lastly, neither empagliflozin nor dapagliflozin independently drove the observed mortality benefit, as meta-regression for both drugs showed no significant interaction with HR (p = 0.7026 for empagliflozin and p = 0.6672 for dapagliflozin). The detailed meta-regression results for all tested moderators are presented in Table S5 (Supplemental material online).

### Randomized controlled trials versus real world evidence

**Fig. 4:**
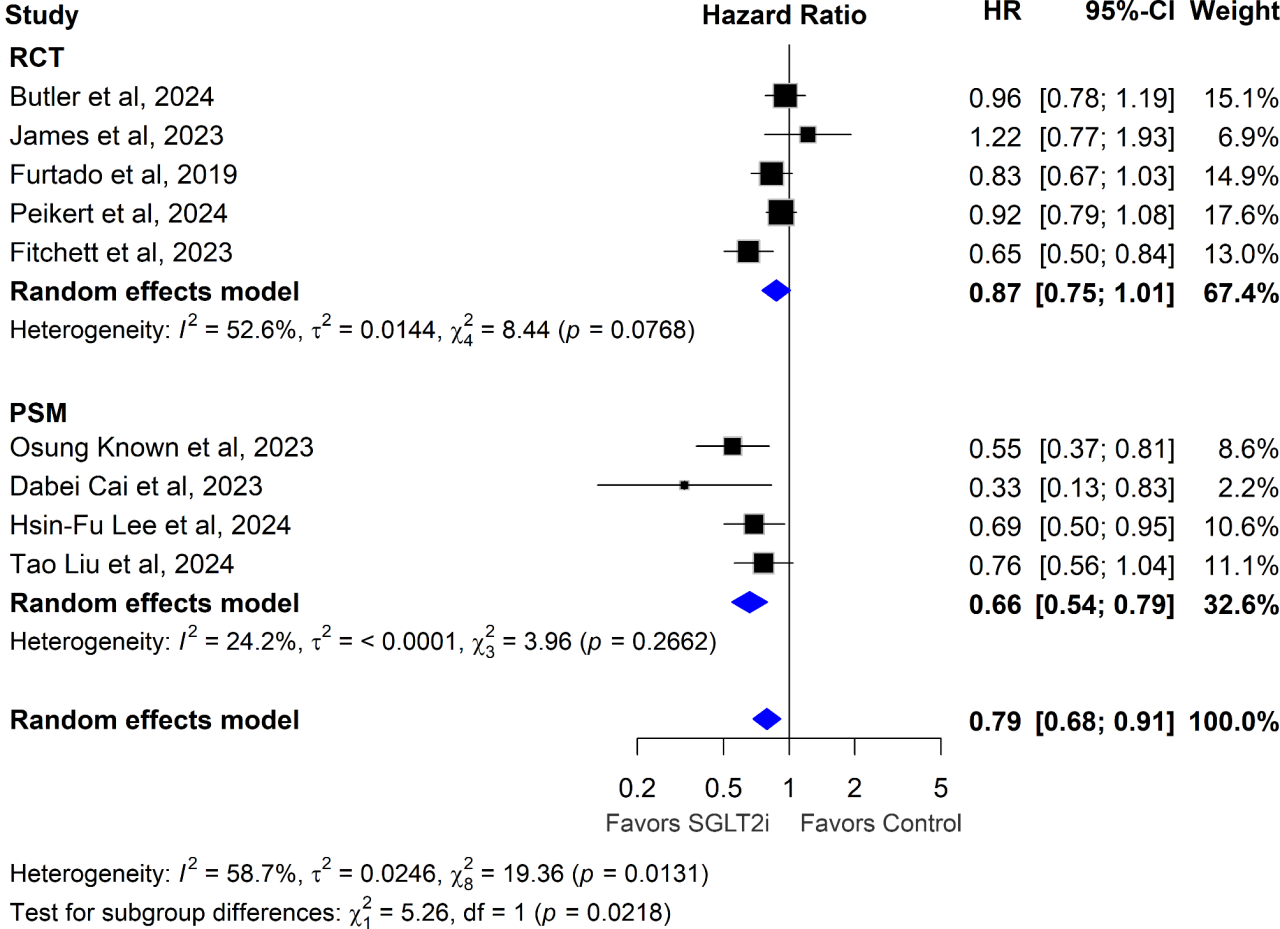
Association of SGLT2 inhibitors with All-Cause Mortality: Analysis Stratified by Study Design (RCT vs PSM). HRs with 95% CIs are plotted, comparing SGLT2 inhibitors to control in post-MI patients, stratified by randomized controlled trials (RCT) and propensity score matched (PSM) studies. HRs below 1 indicate a reduction in all-cause mortality favoring SGLT2 inhibitors

In the RCTs subgroup, SGLT2 inhibitors were associated with a pooled HR for mortality of 0.87 following MI (95% CI: 0.75–1.01), with moderate heterogeneity (I^2^ = 52.6%), as shown in Fig. 4. This indicates a trend toward reduced mortality, though the confidence interval narrowly crossed unity, suggesting the results approached but did not reach statistical significance. Conversely, in the PSM subgroup, SGLT2 inhibitors were significantly associated with reduced mortality, with a pooled HR of 0.66 (95% CI: 0.54–0.79; p < 0.001) and low heterogeneity (I^2^ = 24.2%), as demonstrated in Fig. 4. While RCTs represent the gold standard for causal inference, we hypothesized that the observed differences in treatment associations with mortality could not be fully attributed to study design alone. This hypothesis was supported by sub-group meta-regression analyses, which identified T2DM prevalence as a key moderator of treatment variability. In RCTs, T2DM fully explained the observed heterogeneity (I^2^ = 0), with a significant negative beta coefficient (β = − 0.0041, p = 0.001), indicating that higher proportions of T2DM patients are associated with greater mortality reductions. In contrast, HF status did not significantly explain heterogeneity in RCTs (R^2^ = 0%; β = 0.0012, p = 0.6021). Sensitivity analysis further demonstrated that excluding the DAPA-MI trial, which enrolled no diabetic patients, yielded a significant mortality reduction in RCTs (HR = 0.85, 95% CI: 0.73–0.98; p = 0.03) with reduced heterogeneity (I^2^ = 29.4%), as demonstrated in Fig. S6 (Supplemental material online). PSM studies, by contrast, included predominantly diabetic populations, leading to lower heterogeneity (I^2^ = 24.2%) and stronger associations with reduced mortality.

In the analysis pooling all studies (RCTs and PSM), the combined meta-regression model revealed that T2DM remained a significant moderator (p = 0.01), while the moderating effect of study design was no longer significant after accounting for differences in T2DM distribution (p = 0.29). This demonstrates that the stronger mortality reductions in PSM studies are attributable to the higher prevalence of T2DM rather than the study design itself. Interaction analysis further revealed that the moderating effect of T2DM was consistent across the included studies, with no significant interaction between T2DM and study design (p = 0.12). This indicates that the effect of T2DM on treatment variability is independent of whether the data were derived from RCTs or PSM studies. The complete meta-regression results, including all tested moderators and interactions, are presented in Table S5 (Supplemental material online).

Although PSM studies may introduce residual bias, the higher prevalence of T2DM in these populations helps explain the stronger associations observed between SGLT2 inhibitors and reduced mortality.

### Risk of bias assessment

According to the Cochrane risk-of-bias tool for RCTs (ROB-2), all the RCTs were classified as low risk of bias for the primary outcome of interest (Fig. S8, Supplemental material online). ROBINS-I tool demonstrated a moderate risk of bias for observational studies (Fig. S9, Supplemental material online). Publication bias was assessed by funnel plot visualization (Fig. S10, Supplemental material online). The plot did not show strong evidence of asymmetry, indicating no significant small-study effects. However, due to the limited number of included studies (k = 9), formal statistical testing for funnel plot asymmetry was not feasible.

## Discussion

The principal findings of our study are summarized as follows: (1) SGLT2 inhibitors were associated with a 21% reduction in all-cause mortality post-MI; (2) early treatment following AMI reduced all-cause mortality by 24%; (3) delayed treatment with SGLT2 inhibitors, in conjunction with secondary prevention therapies, led to a 19% reduction in all-cause mortality in patients with a history of MI; (4) meta-regression identified diabetes as a key moderator, with each 1% increase in T2DM patients correlating with a 0.49% further reduction in mortality; (5) HF status was not significantly associated with mortality reduction; (6) there were no significant differences in mortality associations between dapagliflozin and empagliflozin.

Recent studies have demonstrated that empagliflozin reduces the risk of CV outcomes and mortality across a broad spectrum of CV risk in patients with T2DM, including a proportion of patients with prior MI [11]. Conversely, dapagliflozin has shown significant reductions in MACEs in T2DM patients with prior MI, but without consistent effects on mortality [12]. Additionally, Peikert et al. have indicated that dapagliflozin reduces the risk of the composite of CV death and worsening HF in patients with HF and previous MI [13]; however, this study included a minority of individuals with T2DM (49.2%) and failed to demonstrate a mortality reduction.

Patients with a history of MI are at high risk for HHF, regardless of T2DM status. While HF is a leading cause of morbidity and mortality in this population, trials have not consistently shown a linear correlation between reduced HHF rates from SGLT2 inhibitors and mortality.

These findings suggest that while SGLT2 inhibitors consistently improve outcomes such as MACEs and HHF in the post-MI population, their effect on mortality remains inconclusive. This variability may stem from differences in baseline patient characteristics across studies (e.g., proportions of patients with T2DM or HF) or variations in the specific SGLT2 inhibitors. However, our analysis demonstrated a consistent reduction in all-cause mortality with SGLT2 inhibitors in the post-MI population. Notably, T2DM—rather than HF—was the key factor influencing this benefit. Furthermore, our meta-regression revealed no significant differences in mortality association between dapagliflozin and empagliflozin, supporting a class effect for SGLT2 inhibitors post-MI.

Turning to the acute phase of MI, several recent clinical trials have explored the impact of SGLT2 inhibitors when initiated shortly after AMI, yielding conflicting results regarding their effects on NT-proBNP concentrations. While the EMMY and DACAMI trials demonstrated a significant reduction in NT-proBNP levels in patients treated with SGLT2 inhibitors, the SOCOGAMI and EMBODY trials showed no significant decline [14–17]. Moreover, the EMMY trial demonstrated significant differences from baseline in left ventricular ejection fraction and left ventricular end-systolic and end-diastolic volumes in the SGLT2 inhibitors group compared to the placebo group [15]. Notably, the EMMY trial included patients both with and without T2DM, whereas the DACAMI trial excluded those with diabetes.

James et al. [18], in the DAPA-MI study, demonstrated dapagliflozin’s cardiometabolic benefits in AMI patients with impaired left ventricular function and no prior T2DM or chronic HF. However, clinical endpoint rates were lower than expected, and dapagliflozin did not reduce HHF or mortality. The latest EMPACT-MI trial [19] evaluated empagliflozin’s impact on HHF and mortality in patients hospitalized for AMI. Unlike the DAPA-MI, the latter included a higher-risk population, along with a proportion of patients with T2DM (31%). Empagliflozin did not reduce the primary composite endpoint during the trial. However, empagliflozin significantly reduced the hazard of HHF without a significant reduction in mortality.

These studies included varying proportions of individuals with and without T2DM and HF, which contributed to inconsistencies and contrasting results across trials. This variability introduces confusion not due to an issue with SGLT2 inhibitors as a class, but rather due to trial designs that fail to target the most appropriate patient population. Real-world data from PSM observational studies seems to support these findings [20–23]. Targeting a majority of patients with T2DM, these studies consistently showed that early treatment with SGLT2 inhibitors following an AMI favorably impacts CV and mortality outcomes, reinforcing the hypothesis that T2DM is a key factor in achieving the therapeutic benefit.

In our analysis, early treatment with SGLT2 inhibitors following an AMI was associated with a 24% reduction in all-cause mortality, with a growing proportion of diabetics linked to enhanced survival.

A previous meta-analysis of three RCTs and one observational study found that early use of SGLT2 inhibitors in AMI patients treated with PCI reduced HHF risk but had no impact on mortality [33]. Its limitations included a small sample size (n = 1311), short follow-up (23–96 weeks), and the exclusion of recent trials like the DAPA-MI [18] and the EMPACT-MI [19].

Another meta-analysis reported reduced odds of all-cause death and MACEs with SGLT2 inhibitors in ACS patients [34]. However, it included observational studies without PSM, focused on ACS (including unstable angina), and excluded recent RCTs. Neither meta-analysis explored moderators such as T2DM or accounted for differences between trials using meta-regression. In contrast, our meta-analysis specifically focused on post-MI patients, incorporating both RCTs and PSM studies, while implementing a comprehensive meta-regression analysis to elucidate the role of key moderators.

In contemporary practice, advancements in revascularization techniques and background therapies have improved patients’ prognosis following MI. Consequently, the role of cornerstone treatments, such as β-blockers following uncomplicated MI, has been recently questioned [35, 36]. On the other hand, secondary prevention therapies to control cardiometabolic risk factors have been demonstrated to improve clinical outcomes in patients with and without T2DM after CV events [37, 38]. Although our understanding of the mechanism of CV protection with gliflozins following MI remains theoretical, several mechanisms have been proposed [39]. In particular, SGLT2 inhibitors contribute to hemodynamic alterations by lowering pre-load and after-load volumes. These modifications favorably affect ventricular unloading in post-AMI patients, contributing to infarct size reduction [40, 41]. Moreover, SGLT2 inhibitors exhibit potential antioxidant properties that can indirectly interfere with the progression of atherosclerosis and also reduce non-calcified plaque volume [42, 43].

However, our finding of a significant reduction in mortality was not observed in the DAPA-MI [18] or EMPACT-MI [19] trials. Notably, these RCTs were characterized by a low event rate and limited power for mortality endpoints. Additionally, the inclusion of non-diabetic patients in DAPA-MI [18] and the relatively low percentage of diabetic patients (31%) in EMPACT-MI [19] may have contributed to the lack of significant results. In contrast, our findings align with a nationwide observational registry study by H. Christian Rosén, which demonstrated a significant reduction in mortality with SGLT2 inhibitors in patients with T2DM following an AMI [44]. The EMPRESS MI, DAPAPROTECTOR, and PRESTIGE-AMI trials, along with studies by Agban, Gutiérrez, and Li, are evaluating the impact of SGLT2 inhibitors on cardiac remodeling, NT-proBNP levels, and structural changes post-MI [45–49].

Our study underscores the strong association between improved cardiometabolic health and survival following MI, highlighting the effectiveness of SGLT2 inhibitors across the post-MI spectrum and identifying T2DM as a key factor mediating their benefit. While the inclusion of real-world data provides valuable insights into the use of SGLT2 inhibitors in this population, subgroup analysis by study design (RCTs vs. PSM) revealed a more consistent effect in the PSM subgroup. Meta-regression and sensitivity analyses demonstrated that differences in the strength of associations were consistently moderated by T2DM, with its impact extended beyond the study design. However, the inclusion of PSM studies may have introduced residual bias, and reliance on aggregated study-level data limits individual-level insights.

To the best of our knowledge, this is the most extensive systematic review and meta-analysis with meta-regression on SGLT2 inhibitors and mortality post-MI. By stratifying trials into early and delayed treatment, our study adds valuable insights into the timing of SGLT2 inhibitor initiation after MI, which may help refine patient selection and guide future research and practice.

### Study limitations

The authors acknowledge several limitations. First, this meta-analysis with meta-regression relied on aggregated study-level data, which limits granularity and individual-level insights. Second, the inclusion of PSM studies may have introduced bias and confounding. Differences between RCTs and PSM evidence, while both demonstrated the same direction of association with mortality, likely reflect variations in the strength of association, which could be attributed to baseline differences, particularly the proportion of T2DM patients, as highlighted by our exploratory meta-regression and sensitivity analysis. However, the potential for residual bias from PSM studies cannot be completely excluded. Additionally, limitations of the PSM studies included the lack of strict new initiator design (e.g., prior SGLT2 inhibitor exposure in the study by Dabei Cai et al.) and the use of non-active comparators (SGLT2 inhibitors vs. non-users), which may have introduced immortal time bias and residual confounding. On the other hand, PSM studies more closely represent real-world populations, offering valuable insights into the effectiveness of SGLT2 inhibitors in broader clinical settings.

Third, one of the included studies involved patients with unstable angina, representing 0.3% of the overall population. Fourth, one of the six studies classified as an early treatment trial included patients who initiated SGLT2 inhibitors up to 12 weeks after discharge for AMI, partially overlapping with the delayed treatment group. Despite this, excluding this study from both the overall and subgroup analyses did not significantly alter the overall trend toward mortality reduction, as demonstrated by the leave-one-out sensitivity analysis (Figs. S3 and S4, Supplemental material online). Therefore, after careful deliberation among the co-authors, it was decided to retain this study within the early treatment group to preserve the integrity of the overall analysis. Fifth, sensitivity analysis revealed some degree of inconsistencies across subgroups. However, despite these minor variations, the overall trend toward reduced mortality remained consistent, demonstrating the robustness of the analysis.

## Conclusion

In this meta-analysis, early and delayed treatment with SGLT2 inhibitors following MI was associated with a significant reduction in all-cause mortality. Furthermore, the presence of T2DM was associated with a greater mortality reduction, while HF was not significantly associated with the outcome. These findings support the early use of SGLT2 inhibitors in post-MI patients, especially those with T2DM, to improve survival, and suggest that this association may extend beyond HF status. Future RCTs should focus on patients with T2DM to further evaluate the role of SGLT2 inhibitors in the acute MI setting.

## Electronic supplementary material

Below is the link to the electronic supplementary material.

Supplementary Material 1

## Data Availability

No datasets were generated or analysed during the current study.

## Abbreviations

AMI: Acute myocardial infarction
CV: Cardiovascular
HF: Heart failure
HHF: Hospitalization for heart failure
MACEs: Major adverse cardiovascular events
MI: Myocardial infarction
PSM: Propensity score-matched
SGLT2: Sodium–glucose cotransporter-2
T2DM: Type 2 diabetes mellitus
