# Supplementary material for: Association of sodium–glucose cotransporter-2 inhibitors with mortality across the spectrum of myocardial infarction: a systematic review and meta-analysis
Source: Cardiovasc Diabetol. 2025 Jan 22;24:29. doi: 10.1186/s12933-025-02592-0 (PMC11755955; doi:10.1186/s12933-025-02592-0)
Supplement: Supplementary file 1 — Supplementary Material 1 [file 12933_2025_2592_MOESM1_ESM.docx]

M. Maremmani et al. **Association of Sodium-Glucose Cotransporter-2 Inhibitors with Mortality Across the Spectrum of Myocardial Infarction: A systematic review and meta-analysis**

**CONTENTS**

**Table S1**. PRISMA Main Checklist

**Table S2**. PRISMA Abstract Checklist

**Table S3**. Details of the Search Strategy

**Table S4.** Expanded Summaries of the Included Studies

**Fig.** **S1.** Association of SGLT2 inhibitors with HHF: Stratified by RCTs and PSM

**Fig. S2**. Sensitivity Analysis for All-Cause Mortality across the Overall Population Analysis

**Fig. S3.** Sensitivity analysis for All-Cause Mortality in Early treatment trials sub-group analysis

**Fig. S4.** Sensitivity analysis for All-Cause Mortality in Delayed treatment trials sub-group analysis

**Fig. S5.** Sensitivity Analysis for HHF

**Fig.** **S6.** Sensitivity analysis for All-Cause Mortality across Randomized Controlled Trials

**Fig.** **S7.** Sensitivity analysis for All-Cause Mortality across PSM studies

**Table S5.** Meta-regression results

**Fig. S8**. Cochrane’s Risk of Bias Assessment

**Fig. S9**. Risk of Bias Evaluation of Individual Observational Studies by ROBINS-I guidelines.

**Fig. S10**. Funnel Plot evaluating Risk of Publication Bias

**Table S1**. PRISMA Main Checklist

| **Section and Topic** | **Item #** | **Checklist item** | **Location where item is reported** |
| --- | --- | --- | --- |
| **TITLE** | | |  |
| Title | 1 | Identify the report as a systematic review. | Title, Pg. 1 |
| **ABSTRACT** | | |  |
| Abstract | 2 | See the PRISMA 2020 for Abstracts checklist. | Supplemental,  *Table S2* |
| **INTRODUCTION** | | |  |
| Rationale | 3 | Describe the rationale for the review in the context of existing knowledge. | Introduction, Pg. 4-5 |
| Objectives | 4 | Provide an explicit statement of the objective(s) or question(s) the review addresses. | Introduction, Pg. 5 |
| **METHODS** | | |  |
| Eligibility criteria | 5 | Specify the inclusion and exclusion criteria for the review and how studies were grouped for the syntheses. | Methods, Pg.6 |
| Information sources | 6 | Specify all databases, registers, websites, organisations, reference lists and other sources searched or consulted to identify studies. Specify the date when each source was last searched or consulted. | Methods,  Pg.4  Supplemental *Table S3* |
| Search strategy | 7 | Present the full search strategies for all databases, registers and websites, including any filters and limits used. | Methods,  Pg.4;  Supplemental *Table S3* |
| Selection process | 8 | Specify the methods used to decide whether a study met the inclusion criteria of the review, including how many reviewers screened each record and each report retrieved, whether they worked independently, and if applicable, details of automation tools used in the process. | Methods,  Pg.5-6  Supplemental *Table S3* |
| Data collection process | 9 | Specify the methods used to collect data from reports, including how many reviewers collected data from each report, whether they worked independently, any processes for obtaining or confirming data from study investigators, and if applicable, details of automation tools used in the process. | Methods,  Pg.5-6 |
| Data items | 10a | List and define all outcomes for which data were sought. Specify whether all results that were compatible with each outcome domain in each study were sought (e.g. for all measures, time points, analyses), and if not, the methods used to decide which results to collect. | Methods,  Pg.5-6 |
|  | 10b | List and define all other variables for which data were sought (e.g. participant and intervention characteristics, funding sources). Describe any assumptions made about any missing or unclear information. | Methods,  Pg.5-6 |
| Study risk of bias assessment | 11 | Specify the methods used to assess risk of bias in the included studies, including details of the tool(s) used, how many reviewers assessed each study and whether they worked independently, and if applicable, details of automation tools used in the process. | Methods,  Pg. 6 |
| Effect measures | 12 | Specify for each outcome the effect measure(s) (e.g. risk ratio, mean difference) used in the synthesis or presentation of results. | Methods,  Pg. 6 |
| Synthesis methods | 13a | Describe the processes used to decide which studies were eligible for each synthesis (e.g. tabulating the study intervention characteristics and comparing against the planned groups for each synthesis (item #5)). | Methods,  Pg.5-6 |
|  | 13b | Describe any methods required to prepare the data for presentation or synthesis, such as handling of missing summary statistics, or data conversions. | Methods,  Pg.5-6 |
|  | 13c | Describe any methods used to tabulate or visually display results of individual studies and syntheses. | Methods,  Pg.5-6 |
|  | 13d | Describe any methods used to synthesize results and provide a rationale for the choice(s). If meta-analysis was performed, describe the model(s), method(s) to identify the presence and extent of statistical heterogeneity, and software package(s) used. | Methods,  Pg.5-7 |
|  | 13e | Describe any methods used to explore possible causes of heterogeneity among study results (e.g. subgroup analysis, meta-regression). | Methods,  Pg.5-7 |
|  | 13f | Describe any sensitivity analyses conducted to assess robustness of the synthesized results. | Methods,  Pg.5-7 |
| Reporting bias assessment | 14 | Describe any methods used to assess risk of bias due to missing results in a synthesis (arising from reporting biases). | Methods,  Pg.5-7 |
| Certainty assessment | 15 | Describe any methods used to assess certainty (or confidence) in the body of evidence for an outcome. | Methods,  Pg.5-7 |
| **RESULTS** | | |  |
| Study selection | 16a | Describe the results of the search and selection process, from the number of records identified in the search to the number of studies included in the review, ideally using a flow diagram. | Results,  Pg. 8-9;  *Fig. 1* |
|  | 16b | Cite studies that might appear to meet the inclusion criteria, but which were excluded, and explain why they were excluded. | Results,  Pg. 8-9;  *Fig. 1* |
| Study characteristics | 17 | Cite each included study and present its characteristics. | Results,  Pg. 9;  *Table 1* |
| Risk of bias in studies | 18 | Present assessments of risk of bias for each included study. | Results,  Pg. 13-14; Supplement *Fig. S8-S9.* |
| Results of individual studies | 19 | For all outcomes, present, for each study: (a) summary statistics for each group (where appropriate) and (b) an effect estimate and its precision (e.g. confidence/credible interval), ideally using structured tables or plots. | Results,  Pg. 10-11; *Table 1* |
| Results of syntheses | 20a | For each synthesis, briefly summarise the characteristics and risk of bias among contributing studies. | Results,  Pg. 13-14; Supplement *Fig. S8-S9.* |
|  | 20b | Present results of all statistical syntheses conducted. If meta-analysis was done, present for each the summary estimate and its precision (e.g. confidence/credible interval) and measures of statistical heterogeneity. If comparing groups, describe the direction of the effect. | Results,  Pg. 10-11, *Fig. 2-3, Supplement Fig. S1-S7* |
|  | 20c | Present results of all investigations of possible causes of heterogeneity among study results. | Results,  Pg. 12-13; Supplement *Table S5* |
|  | 20d | Present results of all sensitivity analyses conducted to assess the robustness of the synthesized results. | Results,  Pg 11; Supplement *Fig. S2-S3-S4-S5-S6-S7* |
| Reporting biases | 21 | Present assessments of risk of bias due to missing results (arising from reporting biases) for each synthesis assessed. | Results,  Pg. 13-14; Supplement *Fig. S8-S9.* |
| Certainty of evidence | 22 | Present assessments of certainty (or confidence) in the body of evidence for each outcome assessed. | Results,  Pg. 13-14; Supplement *Fig. S8-S9.* |
| **DISCUSSION** | | |  |
| Discussion | 23a | Provide a general interpretation of the results in the context of other evidence. | Discussion, Pg. 14-18 |
|  | 23b | Discuss any limitations of the evidence included in the review. | Study Limitations, Pg.18 |
|  | 23c | Discuss any limitations of the review processes used. | Study Limitations, Pg.18 |
|  | 23d | Discuss implications of the results for practice, policy, and future research. | Discussion, Pg. 14-18  Conclusion,  Pg. 19 |
| **OTHER INFORMATION** | | |  |
| Registration and protocol | 24a | Provide registration information for the review, including register name and registration number, or state that the review was not registered. | Methods,  Pg. 6 |
|  | 24b | Indicate where the review protocol can be accessed, or state that a protocol was not prepared. | Methods,  Pg. 6 |
|  | 24c | Describe and explain any amendments to information provided at registration or in the protocol. | Methods,  Pg. 6 |
| Support | 25 | Describe sources of financial or non-financial support for the review, and the role of the funders or sponsors in the review. | Pg. 20 |
| Competing interests | 26 | Declare any competing interests of review authors. | Pg. 20 |
| Availability of data, code and other materials | 27 | Report which of the following are publicly available and where they can be found: template data collection forms; data extracted from included studies; data used for all analyses; analytic code; any other materials used in the review. | Methods,  Pg. 8; Fig. 1; Table 1; Supplement *Table S4* |

**Table S2**. PRISMA Abstract Checklist

| Topic | No. | Items | Reported? |
| --- | --- | --- | --- |
| TITLE | | | |
| Title | 1 | Identify the report as a systematic review. | Yes |
| BACKGROUND | | | |
| Objectives | 2 | Provide an explicit statement of the main objective(s) or question(s) the review addresses. | Yes |
| METHODS | | | |
| Eligibility criteria  Information sources  Risk of bias  Synthesis of results | 3  4  5  6 | Specify the inclusion and exclusion criteria for the review.  Specify the information sources (e.g. databases, registers) used to identify studies and the date when each was last searched.  Specify the methods used to assess risk of bias in the included studies.  Specify the methods used to present and synthesize results. | Yes  Yes  No  Yes |
| RESULTS | | | |
| Included studies Synthesis of results | 7  8 | Give the total number of included studies and participants and summarise relevant characteristics of studies.  Present results for main outcomes, preferably indicating the number of included studies and participants for each. If meta-analysis was done, report the summary estimate and confidence/credible interval. If comparing groups, indicate the direction of the effect (i.e. which group is favoured). | Yes  Yes |
| DISCUSSION | | | |
| Limitations of evidence  Interpretation | 9  10 | Provide a brief summary of the limitations of the evidence included in the review (e.g. study risk of bias, inconsistency and imprecision).  Provide a general interpretation of the results and important implications. | No  No |
| OTHER | | | |
| Funding  Interpretation | 11  12 | Specify the primary source of funding for the review  Provide the register name and registration number. | No  No |

**Table S3.** Details of the search strategy

| **PubMed** | "Myocardial infarction" OR "Acute MI"OR "Heart attack" OR "Acute coronary syndrome" OR "Acute coronary syndromes" AND "Sodium glucose cotransporter 2 inhibitors" OR "SGLT2" OR "SGLT2 inhibitor” OR "Dapagliflozin" OR "Empagliflozin" OR" Sotagliflozin” OR "Canagliflozin" OR "Ertugliflozin" |
| --- | --- |
| **Embase** | 'heart infarction'/exp OR 'heart infarction' OR 'acute heart infarction' OR 'acute coronary syndrome' AND 'sodium glucose cotransporter 2 inhibitor' OR 'dapagliflozin' OR 'empagliflozin' OR 'canagliflozin' OR 'sotagliflozin' OR 'ertugliflozin' |
| **Scopus** | "Myocardial infarction" OR "Acute MI"OR "Heart attack" OR "Acute coronary syndrome" OR "Acute coronary syndromes" AND "Sodium glucose cotransporter 2 inhibitors" OR "SGLT2" OR "SGLT2 inhibitor” OR "Dapagliflozin" OR "Empagliflozin" OR" Sotagliflozin” OR "Canagliflozin" OR "Ertugliflozin" |
| **Additional sources** | ClinicalTrials.gov  Scientific sessions: American Heart Association (AHA), American College of Cardiology (ACC), ESC congress. |

| **Table S4.** Expanded Summaries of the Included Studies | | | | | | | | | | |
| --- | --- | --- | --- | --- | --- | --- | --- | --- | --- | --- |
| **Name** | **Design** | **Inclusion Criteria** | **Exclusion**  **Criteria** | **Intervention** | **Timing of therapy initiation** | **Arms** | **Mean Age** | **T2DM %**  **(Intervention/Control)** | **Median follow-up** | **Primary**  **Endpoint** |
| James,  2023 | Registry-based, double-blinded, RCT. | AMI and imaging evidence of regional or global impairment  of LV systolic function or definite evidence of Q-wave MI on an EKG. | Established diagnosis of diabetes or chronic HF with LVEF < 40 %. Patients currently on treatment with SGLT2i. | Dapagliflozin 10 mg or placebo once daily. | Within 7-10 days from AMI | Total  (n=4917)  Dapagliflozin  (n=2019)  Placebo (n=1998) | I: 63.0 ±11.06  C: 62.8 ± 10,6 | 0 % (n=0) | 11.6 months | A composite of CV death/hospitalization for HF |
| Butler,  2024 | International,  event-driven, double-blind, RTC. | AMI and either evidence of a newly developed LVEF < 45% or signs or symptoms of congestion and at least one additional enrichment factor. | Previous HF diagnosis or taking/planning to take SGLT2i. | Empagliflozin 10 mg or placebo  once daily. | Within 14 days from AMI | Total  (n=6522)  Empagliflozin (n=3260)  Placebo (n=3262) | I: 63.6 ±11.0  C: 63.7 ± 10.8 | I: 31.7 % (n=1035)  C: 32.1 % (n=1046) | 17.9 months | A composite of hospitalization for heart failure or  death from any cause. |
| Osung Known, 2023 | Observational study using a national health  care service database with a 1:2 PSM | Patients with T2DM treated with PCI for AMI | Patients already on SGLT2i at the index event. | Dapagliflozin (64.5 %)  Empagliflozin (32.2 %)  Ipragliflozin (3.3 %) | Within 14 days after PCI for AMI | Unmatched (n=28671)  Matched 1:2 (n=2814)  I: 938  C: 1876 | I: 56.4 ±11.3  C: 57.6 ± 11.3 | 100 % (2814) | 2.1 years | A composite of hospitalization for heart failure or  death from any cause. |
| Dabei Cai, 2023 | Single center retrospective-cohort with a 1:1 PSM | AMI patients undergone PCI therapy during the hospitalization  accompanied  by T2DM and (or) HFrEF | End-stage kidney disease.  Use of other of SGLT2I during  hospitalization and (or) FU period; use of Dapagliflozin  before admission; initiation of Dapagliflozin after discharge and the FU period; discontinuation of Dapagliflozin during  hospitalization or later. | Dapagliflozin 10 mg vs DAPA-free cohort | During hospitalization for AMI | Unmatched (n=1839)  Matched 1:1 (n=472)  I: 236  C: 236 | I: 62.9±13.0  C: 63.2±12.2 | I: 93.6 % (n=221)  C: 93.6 % (n=221) | 23.8 months | Primary outcome: occurrence of AKI within  7 days after PCI therapy.  Secondary endpoint: all-cause mortality. |
| Hsin-Fu Lee, 2024 | Nationwide, retrospective, cohort study  With 1:1 PSM | T2DM patients after AMI | End-stage renal disease.  Treated with SGLT2i before AMI.  Treated with  SGLT2i after 3 months from the date of AMI.  Those who did not receive any diabetic medications.  FU < 6 months. | Empagliflozin (57%)  Dapagliflozin (38%)  Canagliflozin (5%) | Within 3 months after discharge for AMI | Unmatched (n=9885)  Matched 1:1 (n=1888)  I: 944  C: 944 | I: 63.1 ± 11.9  C: 63.9 ± 11.3 | I: 100 % (n=944)  C: 100 % (n=944) | I: 1.96 ± 1.09 years ^c^  C: 1.91 ± 1.12 years ^c^ | Cardiovascular Death  Hospitalization for HF  All-cause death  MACE  Myocardial Infarction  Coronary Revascularization  Ischemic stroke |
| Tao Liu, 2024 | Single-center retrospective study with 1:1 PSM | Hospitalized for ACS and T2DM | Previous use of  SGLT2i.  Cardiogenic shock Severe HF  (Killip class ≥ III). Malignant ventricular arrhythmias.  Severe hepatic or renal dysfunction (eGFR < 30 ml/min/1.73 m2).  Perioperative cardiopulmonary  Resuscitation. Malignant tumors. | SGLT2i  (specific SGLT2i non reported) | During hospitalization for ACS | Unmatched (n=925)  Matched 1:1 (n=452)  I: 226  C: 226 | I: 62.9 ± 10.8  C: 62.1 ± 11.7 | I: 100 % (n=226)  C: 100 % (n=226) | 12 months | MACE  CV death  All-cause death  nonfatal myocardial infarction  Stroke  Coronary revascularization  HF readmission. |
| Furtado, 2019 | Prespecified sub-group analysis of randomized, double-blind, placebo-controlled trial | T2DM and established ASCVD or MRF for atherosclerotic CV disease > prespecified sub-group of patients with a history of MI | Acute CV event  Un-controlled hypertension  T1D  Malignancy  eGFR < 60 ml/min  Previous treatment with SGLT2i | Dapagliflozin 10 mg or placebo  once daily. | Not earlier than 8 weeks from an acute CV event | Total  (n=3584)  Dapagliflozin (n=1777)  Placebo (n=1807) | I: 62.0 ± 7.9^a^  C: 62.0 ± 7.9^a^ | I: 100 % (n=1777)  C: 100 %(n=1807) | 50.4 months | A composite of MACE (cardiovascular death, MI, or ischemic  stroke) and the composite of cardiovascular death or hospitalization for heart failure. |
| Peikert, 2024 | Participant-level pooled analysis of RCTs | Planned participant level pooled analysis: prior MI sub-group.  DAPA-HF: NYHA II-IV and LVEF ≤ 40 % and laboratory parameter  DELIVER-HF: NYHA II-IV and LVEF > 40 % and laboratory parameter | DAPA-HF: T1DM,  symptoms of hypotension or a systolic BP < 95 mm Hg, and eGFR < 30 ml/min  DELIVER-HF: SGLT2i within 4 weeks, T1D, eGFR < 25 ml/min, infiltrative cardiomyopathy, uncontrolled hypertension, valvular disease. | Dapagliflozin 10 mg or placebo  once daily. | Not earlier than 12 weeks from an acute CV event | Total  (n=3731)  Dapagliflozin (n=1830)  Placebo (n=1901) | I/C: 63.1 ± 8.6^b^ | I/C: 49.2 % (n=1835)^b^ | DAPA-HF: 18.2 months  DELIVER:27.6 months | A composite  of worsening heart failure (hospitalization or an urgent visit resulting in intravenous  therapy for heart failure) or cardiovascular death. |
| Fitchett, 2019 | Prespecified sub-group analysis of randomized, double-blind, placebo-controlled trial  (1:1:1) | T2DM and established cardiovascular disease and had a glycated hemoglobin level of at least 7.0% and no more than 9.0%.  eGFR ≥ 30 ml/min | ACS, stroke, or transient ischemic attack within 2 months.  SGLT2i treatment within 30 days  eGFR < 30 ml/min  Uncontrolled Hyperglycemia | Empagliflozin 10 mg or 25 mg or  placebo once daily. | Not earlier than 8 weeks from an acute CV event | Total  (n=3273)  Empagliflozin (n=2190)  Placebo (n=1083) | I: 63.1 ± 8.6  C: 63.2 ± 8.8 | I: 100 % (n=2190)  C: 100 %  (n=1083) | 37.2 months | A composite outcome was death from cardiovascular  causes, nonfatal myocardial infarction, or nonfatal stroke |
| Values are mean ± SD, n (%), or median. ^a^Median=Mean and IQR=SDx1.38. ^b^Specific values for intervention and control groups were not reported. ^c^Follow-up duration reported as mean ± SD  Abbreviations: ACS, acute coronary syndrome; AMI, acute myocardial infarction; ASCVD, atherosclerotic cardiovascular disease; C, control cohort; CV, cardiovascular; eGFR, estimated glomerular filtration rate; FU, follow-up; HF, heart failure; I, intervention cohort; LVEF, left ventricular ejection fraction; MRF, multiple risk factors; PCI, percutaneous coronary intervention; PSM, propensity score matching; RCT, randomized controlled trial; SGLT2i, sodium-glucose cotransporter 2 inhibitor; T2DM, type 2 diabetes mellitus. | | | | | | | | | | |

**
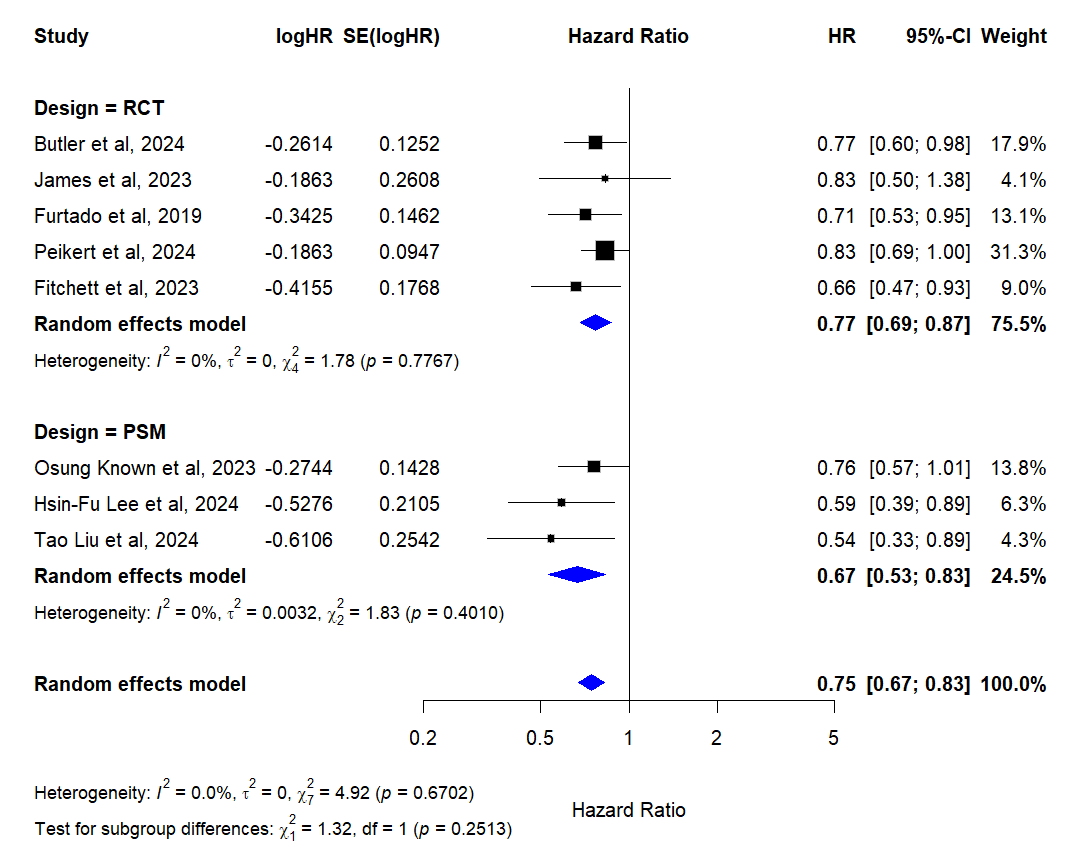
Fig.** **S1.** Association of SGLT2 inhibitors with HHF: stratified by RCTs and PSM

**Fig. S2.** Sensitivity Analysis for All-Cause Mortality across the Overall Population analysis

**
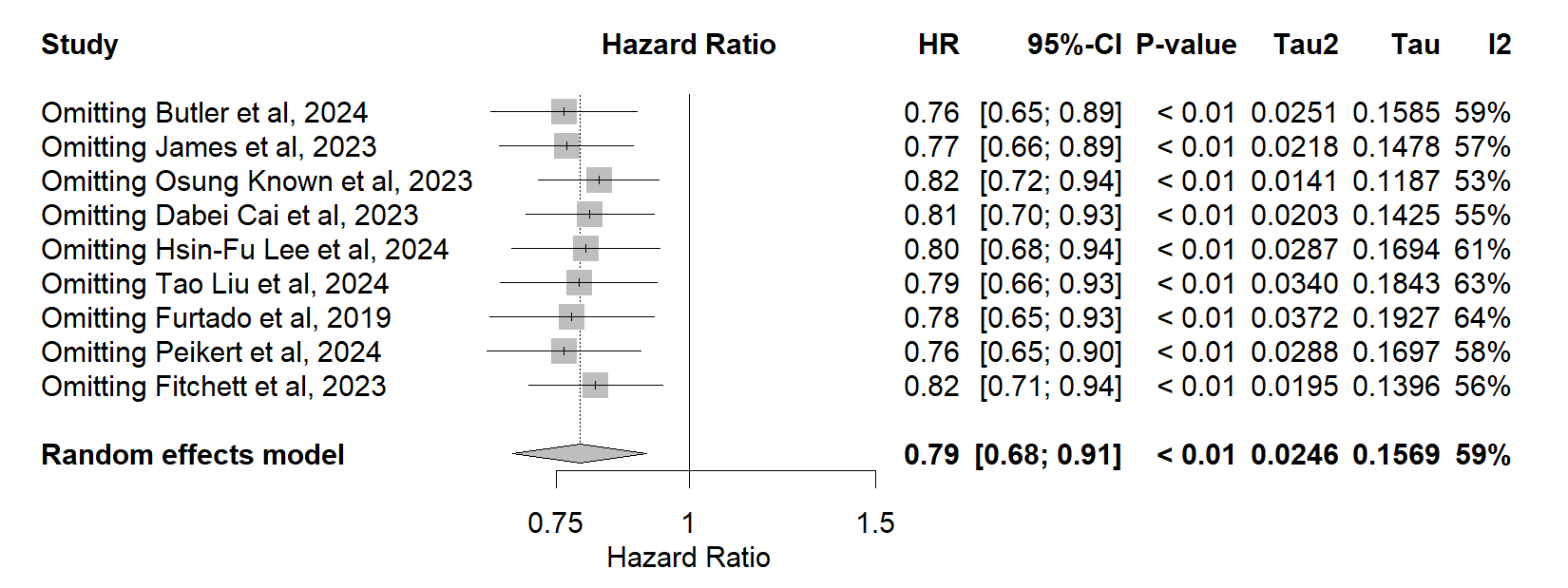
**

**Fig. S3.** Sensitivity analysis for All-Cause Mortality in Early treatment trials sub-group analysis

**
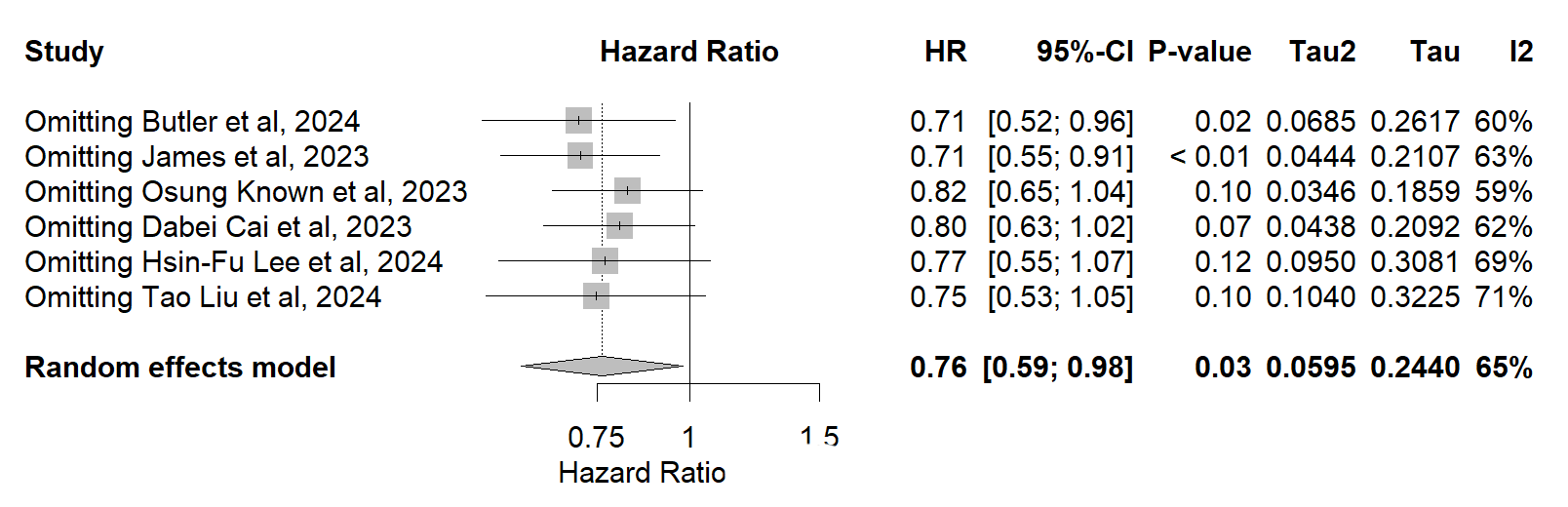
**

**Fig. S4.** Sensitivity analysis for All-Cause Mortality in Delayed treatment trials sub-group analysis

**
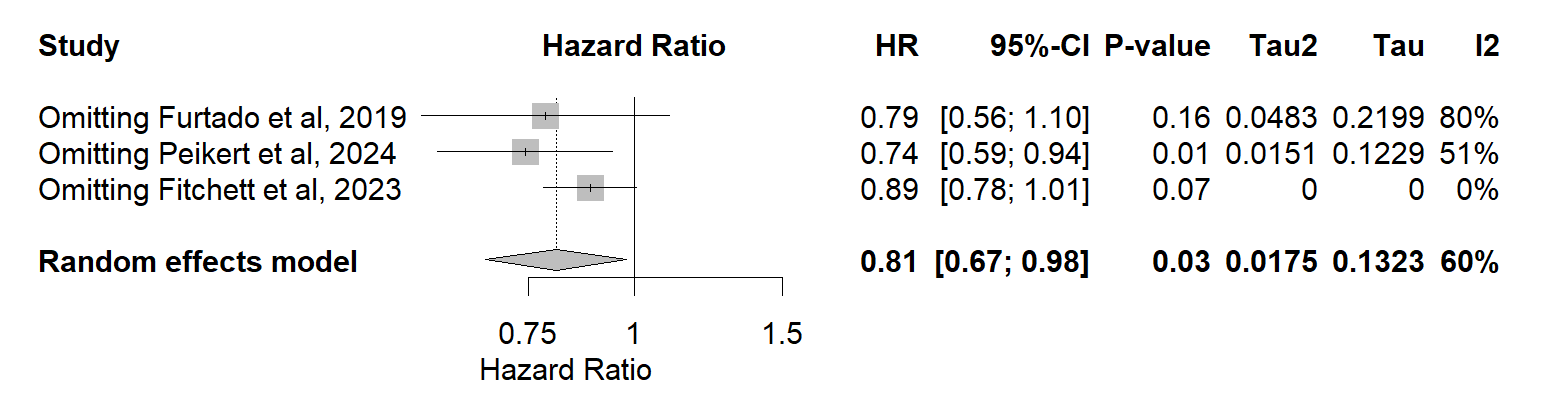
**

**
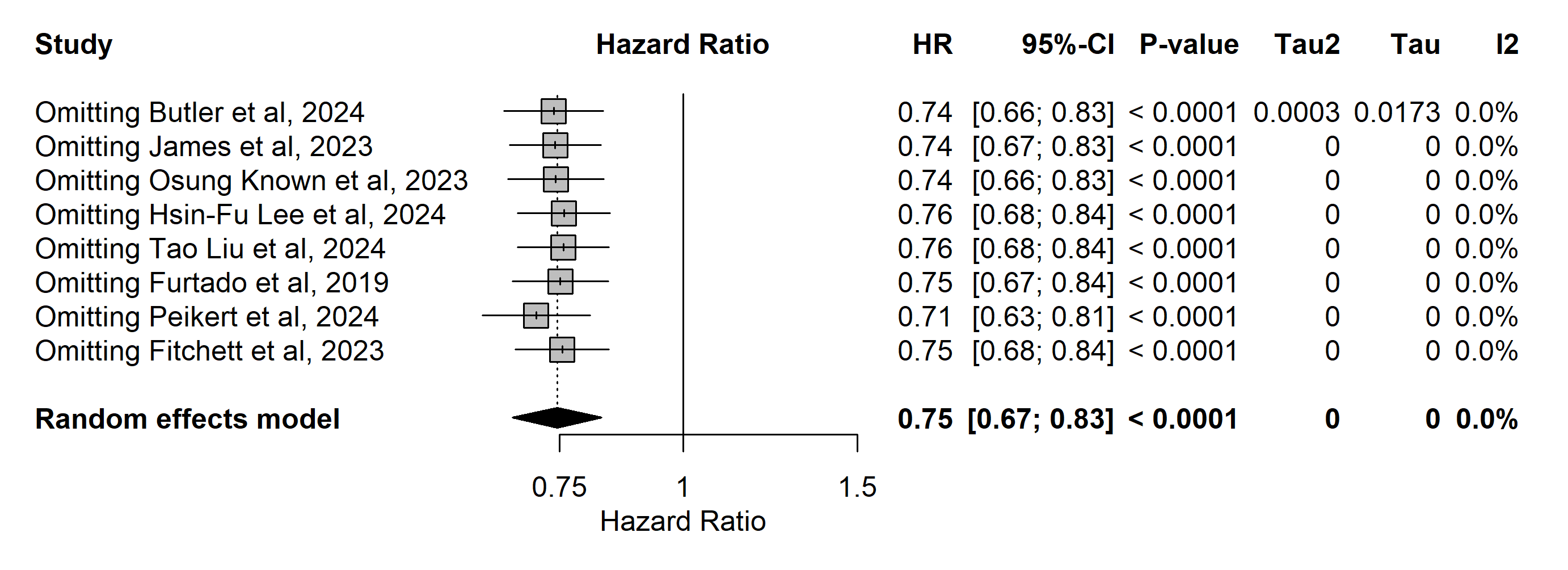
Fig. S5.** Sensitivity Analysis for HHF

**Fig.** **S6.** Sensitivity analysis for All-Cause Mortality across Randomized Controlled Trials
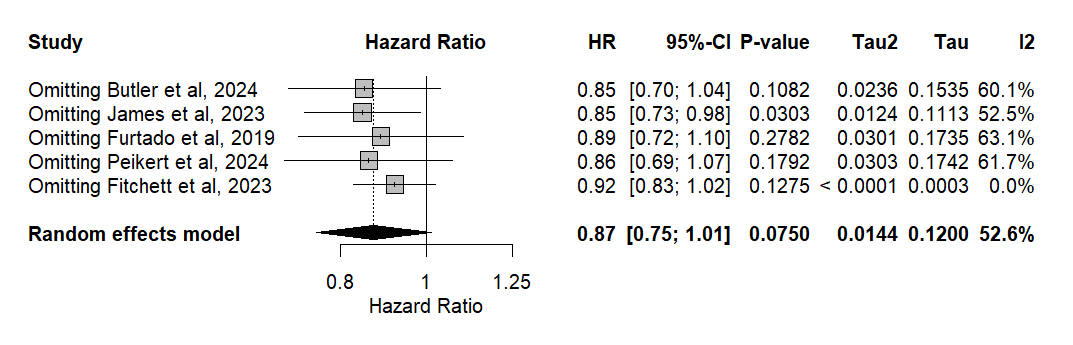


**Fig.** **S7.** Sensitivity analysis for All-Cause Mortality across PSM studies
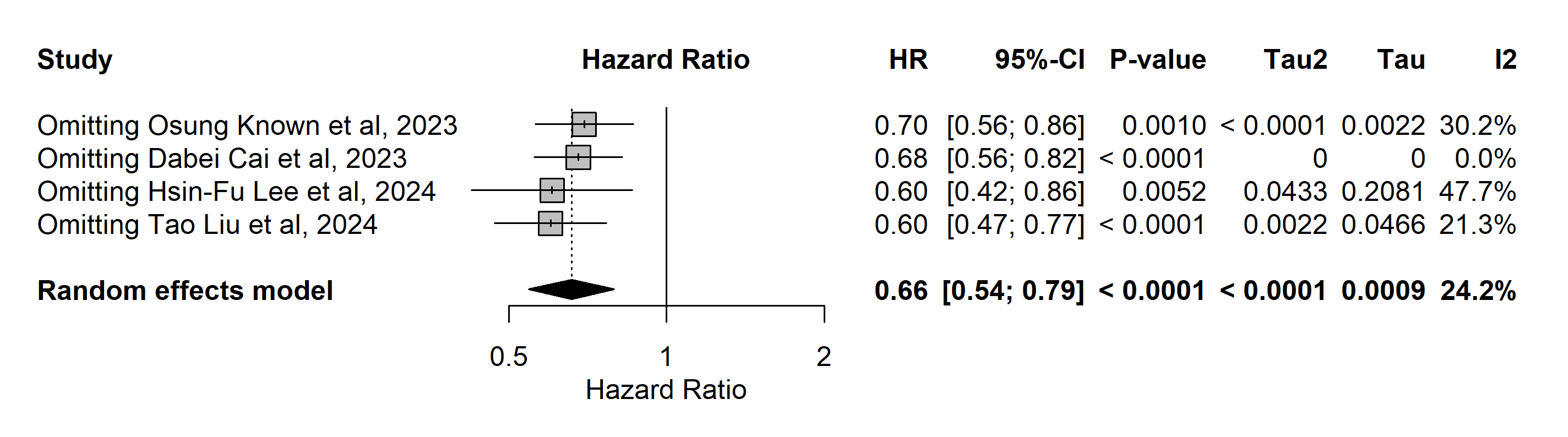


**Table S5.** Meta-regression results

| **Moderators (%)** | **Tau²** | **I² (%)** | **R² (%)** | **Intercept (Estimate, p-value)** | **Moderator**  **(β-coefficient, p-value)** |
| --- | --- | --- | --- | --- | --- |
| **Overall Trials (K=9)** |  |  |  |  |  |
| Type 2 diabetes mellitus (%) | 0.0000 | 0.00 | 100.00 | 0.1440, p = 0.1797 | -0.0049, p = 0.0006 |
| Heart failure (%) | 0.0098 | 30.34 | 60.20 | -0.3412, p = 0.0001 | 0.0030, p = 0.0705 |
| Empagliflozin (%) | 0.0340 | 61.65 | 0.00 | -0.2157, p = 0.0441 | -0.0007, p = 0.7026 |
| Dapagliflozin (%) | 0.0334 | 61.27 | 0.00 | -0.2922, p = 0.0401 | 0.0008, p = 0.6672 |
| Study design (RCTs vs PSM)† | 0.0113 | 37.30 | 54.04 | \|  \| \| --- \|  \| -0.1369, p = 0.0575 \| \| --- \| | -0.2972, p = 0.0267 |
| Combined model (T2DM + study design†) | 0.0000 | 0.00 | 100.00 | 0.1126, p = 0.3123 | **T2DM:** -0.0040, p = 0.013 **Study Design:** -0.1312, p = 0.295 |
| Interaction between T2DM and study design† | / | / | / | / | 0.1166, p = 0.1201 |
| **Early Treatment trials**  **(K=6)** |  |  |  |  |  |
| Type 2 diabetes mellitus (%) | 0.0000 | 0.02 | 99.99 | 0.1531, p = 0.2551 | -0.0057, p = 0.0016 |
| Heart failure (%) | 0.0823 | 68.59 | 0.00 | -0.3662, p = 0.0715 | 0.0037, p = 0.5522 |
| **Delayed Treatment trials (K=3)** |  |  |  |  |  |
| Type 2 diabetes mellitus (%) | 0.0151 | 50.55 | 13.66 | 0.1238, p = 0.6918 | -0.0042, p = 0.2622 |
| Heart failure (%) | 0.0093 | 39.32 | 46.61 | -0.3481, p = 0.0095 | 0.0027, p = 0.1740 |
| **Randomized controlled trials (K=5)** |  |  |  |  |  |
| Type 2 diabetes mellitus (%) | 0.0000 | 0.00 | 100.00 | 0.1160, p = 0.2982 | -0.0041, p = 0.012 |
| Heart failure (%) | **0.0214** | 57.29 | 0.00 | -0.1917, p= 0.1661 | 0.0012, p = 0.6021 |
| **Propensity score matching (K=4)** |  |  |  |  |  |
| Type 2 diabetes mellitus (%) | 0.0000 | 0.00 | 100.00 | -11.6410, p = 0.1197 | 0.1125, p = 0.13 |
| Heart failure (%) | 0.0011 | 1.97 | 0.00 | -0.5220, p = 0.0002 | β = 0.0057, p = 0.3118 |
| K=number of comparisons  †Moderator has been categorized as a binary variable, with RCTs coded as 0 and PSM studies coded as 1. | | | | | |

**Fig. S8.** Cochrane’s Risk of Bias Assessment

1. *Cochrane’s Risk of Bias assessment graph*


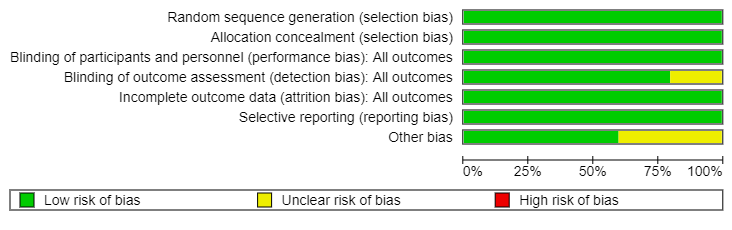


1. *Cochrane’s Risk of Bias assessment summary.*


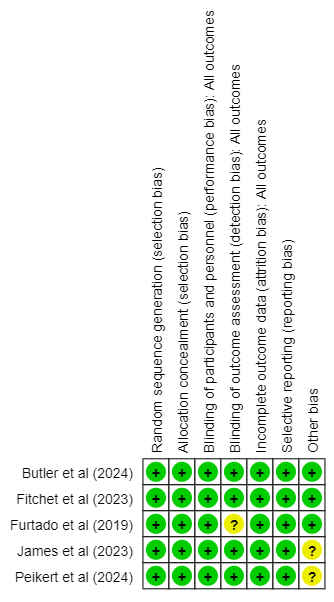


**Fig. S9.** Risk of Bias Evaluation of Individual Observational Studies by ROBINS-I guidelines

|  | Tao Liu 2024  (non-RCT) | Hsin-Fun Lee 2024  (non-RCT) | Osung Known 2023  (non-RCT) | Dabei Cai  2023 (non-RCT) |
| --- | --- | --- | --- | --- |
| Bias due to confounding | 1.1 Y; 1.2 PN; 1.4 Y; 1.5 PY; 1.6 PN; 1.7 NA  Moderate risk | 1.1 Y; 1.2 PN; 1.4 Y; 1.5 PY; 1.6 PN; 1.7 NA  Moderate risk | 1.1 Y; 1.2 PN; 1.4 Y; 1.5 PY; 1.6 PN; 1.7 NA  Moderate risk | 1.1 Y; 1.2 PN; 1.4 Y; 1.5 PY; 1.6 PN; 1.7 NA  Moderate risk |
| Bias in the selection of participants into the study | 2.1 Y; 2.2 N; 2.4 Y  Moderate risk | 2.1 Y; 2.2 N; 2.4 Y  Moderate risk | 2.1 Y; 2.2 N; 2.4 Y  Moderate risk | 2.1 Y; 2.2 N; 2.4 Y  Moderate risk |
| Bias in the classification of interventions | 3.1 Y; 3.2 PN; 3.3 PN  Moderate Risk | 3.1 Y; 3.2 PN; 3.3 PN  Moderate Risk | 3.1 Y; 3.2 PN; 3.3 PN  Moderate Risk | 3.1 Y; 3.2 PN; 3.3 PN  Moderate Risk |
| Bias due to deviations from intended interventions | 4.1 PN; 4.3 PY; 4.4 PY; 4.5 Y  Low risk | 4.1 PN; 4.3 PY; 4.4 PY; 4.5 Y  Low risk | 4.1 PN; 4.3 PY; 4.4 PY; 4.5 Y  Low risk | 4.1 PN; 4.3 PY; 4.4 PY; 4.5 Y  Low risk |
| Bias due to missing data | 5.1 PY; 5.2 PN; 5.3 PN  Low Risk | 5.1 PY; 5.2 PN; 5.3 PN  Low Risk | 5.1 PY; 5.2 PN; 5.3 PN  Low Risk | 5.1 PY; 5.2 PN; 5.3 PN  Low Risk |
| Bias in the measurement of outcomes | 6.1 N; 6.2 PY; 6.3 PY; 6.4 PN  Low risk | 6.1 N; 6.2 PY; 6.3 PY; 6.4 PN  Low risk | 6.1 N; 6.2 PY; 6.3 PY; 6.4 PN  Low risk | 6.1 N; 6.2 PY; 6.3 PY; 6.4 PN  Low risk |
| Bias in the selection of the reported result | 7.1 PN; 7.2 PN; 7.3 PN  Low risk | 7.1 PN; 7.2 PN; 7.3 PN  Low risk | 7.1 PN; 7.2 PN; 7.3 PN  Low risk | 7.1 PN; 7.2 PN; 7.3 PN  Low risk |
| Overall bias | Moderate Risk | Moderate Risk | Moderate Risk | Moderate risk |

Yes (Y), Probably yes (PY), Probably no (PN), No (N), Not appliable (NA), No information (NI).

**
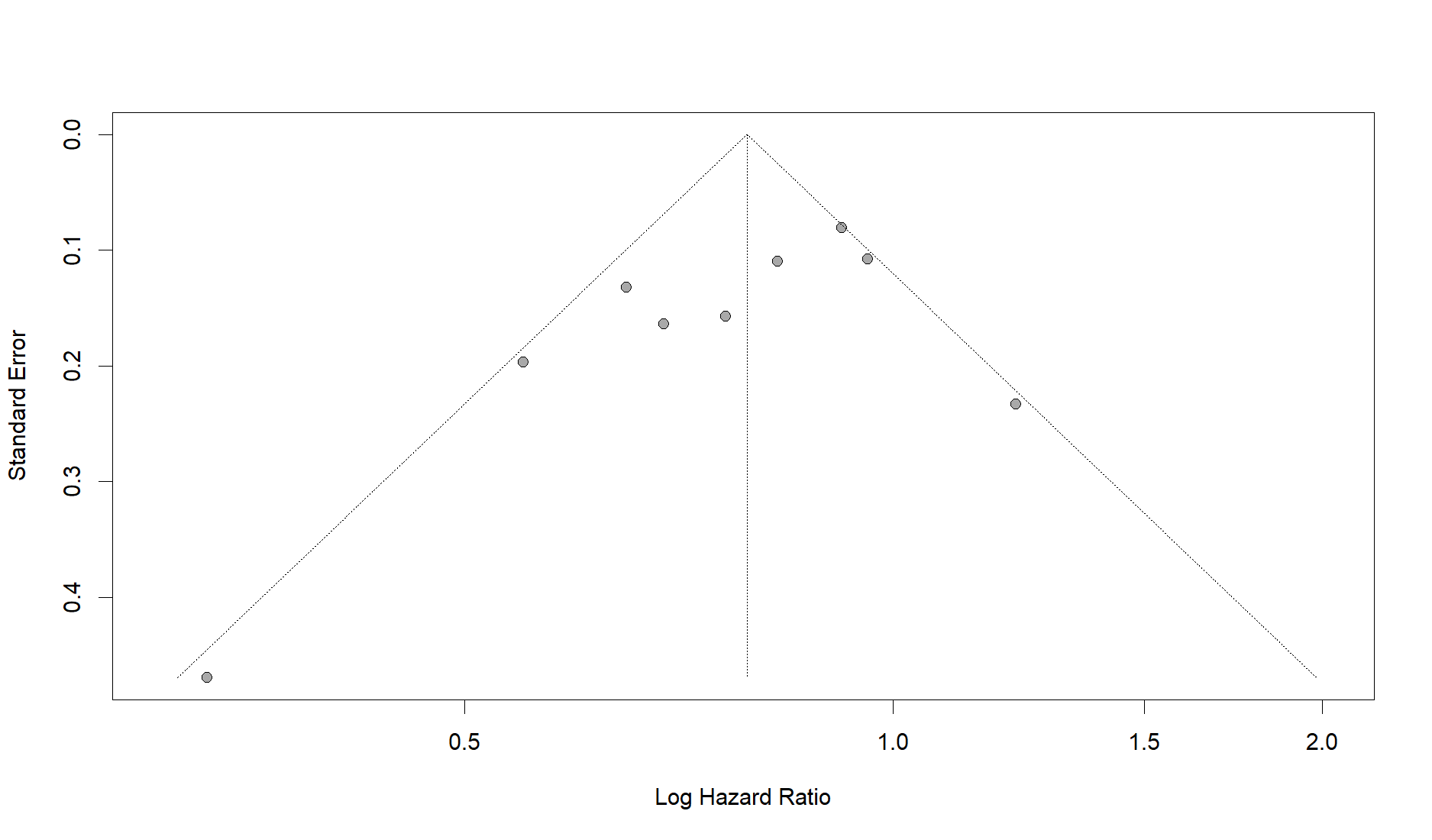
Fig. S10.** Funnel Plot evaluating Risk of Publication Bias
